# Assessing the intraspecific osteological variation of the spottail shiner ( *Hudsonius hudsonius* ) (Cypriniformes: Leuciscidae)

**DOI:** 10.1111/jfb.70107

**Published:** 2025-06-11

**Authors:** Erika K. Jessen

**Affiliations:** ^1^ Department of Biology McGill University Montreal Quebec Canada

**Keywords:** *Hudsonius hudsonius*, intraspecific variation, notropin fishes, osteology, regional differentiation, spottail shiner

## Abstract

*Hudsonius hudsonius* (Cypriniformes: Leuciscidae), commonly known as the spottail shiner, is a small cyprinoid fish species found across much of North America. *H. hudsonius* has traditionally been regarded as one of the most basal and plesiomorphic species among the notropin fishes, that is, fishes traditionally placed in or associated with the large, polyphyletic genus *Notropi*s, including but not limited to *Alburnops*, *Cyprinella*, *Ericymba*, *Lythrurus*, *Miniellus* and *Paranonotropis*. The basal nature of *H. hudsonius* has been further corroborated by recent molecular studies. The osteology of notropin fishes has either focused on a specific region of the skeleton, including the cranium, pharyngeal teeth and dental formulae, or the caudal skeleton. Others have aimed to determine evolutionary relationships using osteological characteristics. However, no research has focused on the osteology of *H. hudsonius* specifically nor emphasized the intraspecific osteology of a single notropin species. Thus, this study aimed to describe and analyse key osteological characters of *H. hudsonius*, highlighting key osteological variants within and among 15 populations from Alberta, the Northwest Territories, Manitoba and Ontario. Particular attention is given to the elements of the oral jaws, suspensorium, opercular series, branchial apparatus, hyoid region, circumorbital series, pectoral girdle, pelvic girdle, caudal skeleton and skull. My analysis of the osteology of *H. hudsonius* reveals variation in all examined regions except the pelvic girdle. Regional differentiation between eastern and western populations is especially evident in the degree of fusion of the pectoral girdle, fusion of the parhypural and first hypural of the caudal skeleton and shape of the urohyal. The results of this analysis provide an overview of the osteology of a basal notropin species and highlight which regions are subject to variation. It also raises further questions about the current taxonomic classification of *H. hudsonius* based on the regional differences observed here.

## 1 INTRODUCTION

*Hudsonius hudsonius* Clinton, 1824, commonly known as the spottail shiner, is a cyprinoid fish species native to North America. It is widely distributed from the eastern United States and as far north as the Northwest Territories in Canada (Froese & Pauly, 2023; Page & Burr, 2011). They are small fish, reaching no more than 147 mm in total length (Gidmark & Simons, 2014). *H. hudsonius* is found in various freshwater habitats, including lakes, streams and rivers, and has an omnivorous diet (Gidmark & Simons, 2014; Nelson & Paetz, 1992). Distinguishing features of *H. hudsonius* include a moderately compressed body, blue green colouring dorsally and silver colouring laterally. The most distinct marking is a large black dot at the base of the caudal fin (Page & Burr, 2011).

In his analysis of the osteology of *Notropis atherinoides* Rafinesque, 1818 and 77 other *Notropis* Rafinesque, 1818 (s.l.) species, Coburn (1982) considered *H. hudsonius* to be among the most plesiomorphic members of the genus as it was then defined, based on several morphological and behavioural characteristics reflecting a generalist lifestyle. Although he conducted phylogenetic analyses for some taxa, *H. hudsonius* was not included and was not assigned a sister species. Since then, molecular studies have provided phylogenomic support for the basal nature of *H. hudsonius*. Using sequences from the mitochondrial cytochrome b gene, Mayden et al. (2006) assessed the phylogenetic relationships of 168 different notropin shiner species. The resulting analysis placed *H. hudsonius* within the basal, monophyletic *Hudsonius* Girard, 1856 clade, in which *H. hudsonius* was sister to a clade containing *Hudsonius altipinnis* Cope, 1870 and *Pteronotropis cummingsae* Meyers, 1925. *Hudsonius* formed a trichotomy with a group of three species from the polyphyletic genus *Pteronotropis* Fowler, 1935, and a large group the authors dubbed the ‘notropin clade’, encompassing a vast majority of genera/species traditionally placed within *Notropis* (s.l.). Although the monophyly of Mayden et al.'s (2006) *Hudsonius* clade has faced some challenges (Schönhuth et al., 2018; Stout et al., 2022), the basal placement of *H. hudsonius* has been consistently supported (Mayden et al., 2006; Schönhuth et al., 2018; Stout et al., 2022).

Past research on the osteology of *H. hudsonius* has been in the context of wider phylogenetic studies (Coburn, 1982; Mayden, 1989) or studies focusing on a specific osteological characteristic among other North American cyprinoids, like the caudal skeleton or pharyngeal teeth (Buhan, 1972; Eastman & Underhill, 1973). Despite the basal nature of *H. hudsonius*, research has yet to focus and describe its osteology specifically. Furthermore, research has yet to focus on the intraspecific osteological variation in *H. hudsonius* or other notropin species. This paper describes osteology of *H. hudsonius* and identifies osteological features subject to variability. By describing the osteology of this taxon, I will not only outline the osteology of a basal, plesiomorphic notropin species but also determine what parts of the skeleton of *H. hudsonius* are subject to variability and reveal any patterns of this variation that may exist among different populations.

## 2 MATERIALS AND METHODS

### 2.1 Ethics statement

Ethical and care considerations were not required for this study, as it exclusively used museum specimens preserved in ethanol or glycerin, all of which were collected prior to the study's commencement. No live animals were handled or involved in any part of this research.

### 2.2 Materials

The *H. hudsonius* specimens used in this study are catalogued in the University of Alberta Museum of Zoology Ichthyology Collection, Edmonton, Canada, and the University of Manitoba Ichthyology Collection, Winnipeg, Canada. I used a total of 72 specimens sampled from 15 different populations in Alberta (11), Manitoba (2), western Ontario (1) and the Northwest Territories (1). Four population samples from the University of Alberta's collections were cleared and stained prior to this study and stored in 100% glycerin. All remaining specimens were initially preserved in alcohol and then cleared and stained following the protocols of Taylor and Van Dyke (1985).

### 2.3 Measurements and analysis

Measurements (*n* = 54), meristic counts (*n =* 54) and dental formulas (*n* = 43) were documented according to Hubbs and Lagler (2004). Scale counts were collected according to Armbruster (2012), whereas pre‐anal, pre‐pectoral and pre‐pelvic lengths were measured following Habib et al. (2019). Vertebral counts (*n* = 42) included the four Weberian centra and the compound centrum of the caudal skeleton (Fink & Fink, 1981). Pore counts (*n* = 24) of the supraorbital, supratemporal, infraorbital, preopercular mandibular and postocular commissure sensory canals were taken from cleared and stained specimens and counted according to Illick (1956).

A total of 50 cleared and stained specimens were analysed. Photographs for osteological drawings were taken with a Zeiss Stereo Discovery.V8 stereomicroscope with a Carl Zeiss 44,403 6‐9000 eyepiece (8×) and a variety of lenses (Zeiss Achromat S 0.3× FWD 236 mm, Zeiss Plan Apo S 0.63× FWD 81 mm and Zeiss Plan Apo S 1.0× FWD 60 mm), with NIS‐Elements F package 2.20, version 5.03. Osteological terminology generally follows Coburn (1982) and Conway (2011) unless otherwise specified. Terminology relating to the sensory canal system follows Reno (1966). A total of 20 cleared and stained specimens were further dissected, having their branchial apparatuses removed. All foramina are identified following Coburn (1989) and Conway (2011). My analysis focused on the oral jaws (*n* = 45), suspensorium (*n* = 45), opercular series (*n* = 39), branchial apparatus (*n* = 22), hyoid region (*n* = 46), circumorbital series (*n* = 39), Weberian apparatus (*n* = 49), pectoral girdle (*n* = 45), pelvic girdle (*n* = 45) and caudal skeleton (*n* = 50). I also analysed the elements of the skull, breaking my analysis into an ethmoid region (*n* = 49), orbital region (*n* = 49), otic and occipital region (*n* = 20) and basicranial region (*n* = 20). My analysis of the branchial apparatus, otic, occipital and basicranial regions was limited by the number of specimens I was able to dissect.

## 3 RESULTS

### 3.1 Ethmoid region

**FIGURE 1:**
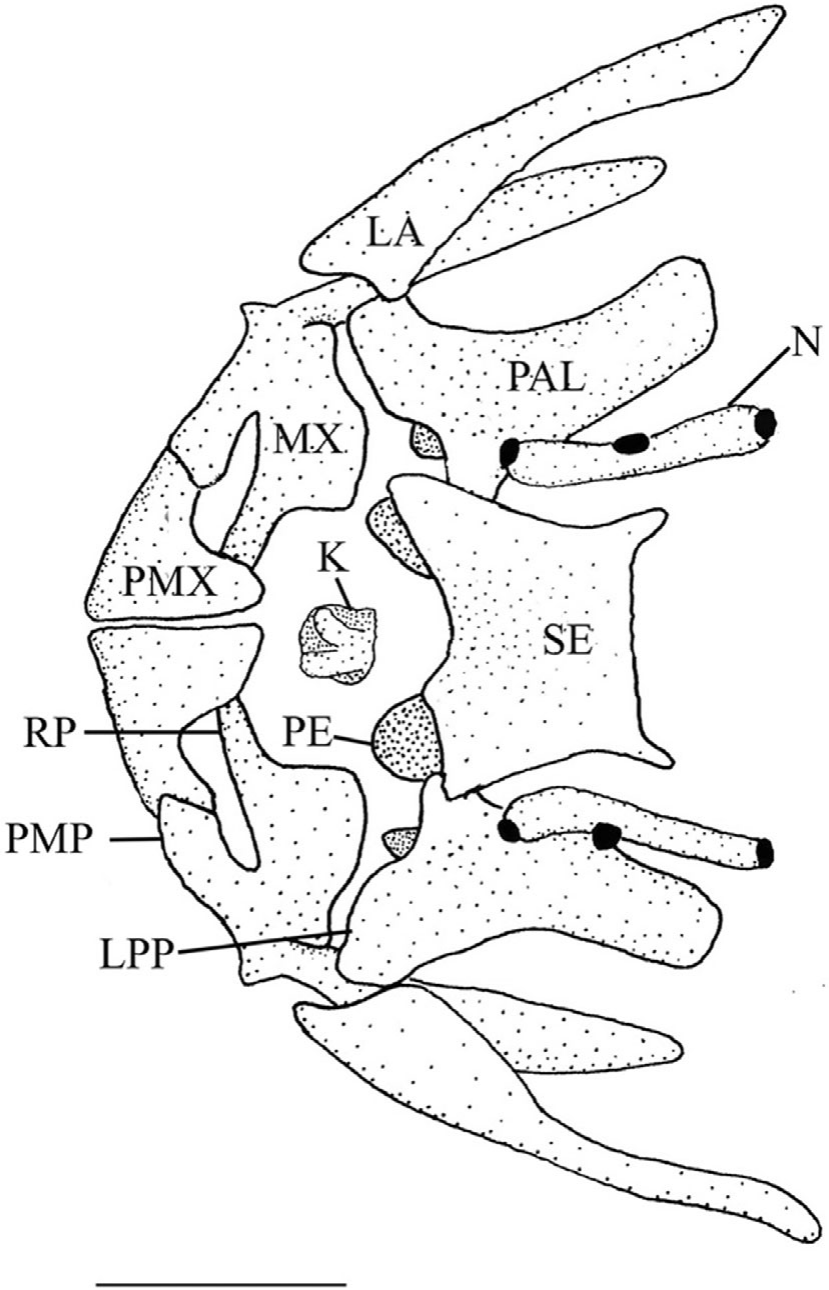
Illustration of the supraethmoid, nasals, maxillae, premaxillae and kinethmoid of *Hudsonius hudsonius* UAMZ F1258, specimen 1, in dorsal view, anterior to top of the page. The mouth is closed in this illustration. K, kinethmoid; LA, lacrimal; LPP, lateral process of the palatine; MX, maxilla; N, nasal; PAL, palatine; PE, preethmoid; PMP, premaxillary process of the premaxilla; PMX, premaxilla; RP, rostral process of the maxilla; SE, supraethmoid portion of the ethmoid block. Scale bar = 1 mm.

The ethmoid block or complex is a single, median element that makes up the anterior portion of the nasal cavity. According to Conway (2011), the ethmoid block has a fused mesethmoid (ME) and supraethmoid portion (SE). It has a deep anterior notch that accommodates the kinethmoid (K) and an expanded base that sits on a cartilage strip, the planum ethmoidale (Harrington, 1955), separating it from the vomer (VO). The mesethmoid portion forms the ventral part of the ethmoid complex. It is oval and constricted in the middle, forming a nasal septum. The supraethmoid portion forms the dorsal region of the ethmoid complex, roofing the nasal cavity. It is dorsally flattened and appears constricted towards the posterior end in dorsal view (Figure 1).

**FIGURE 2:**
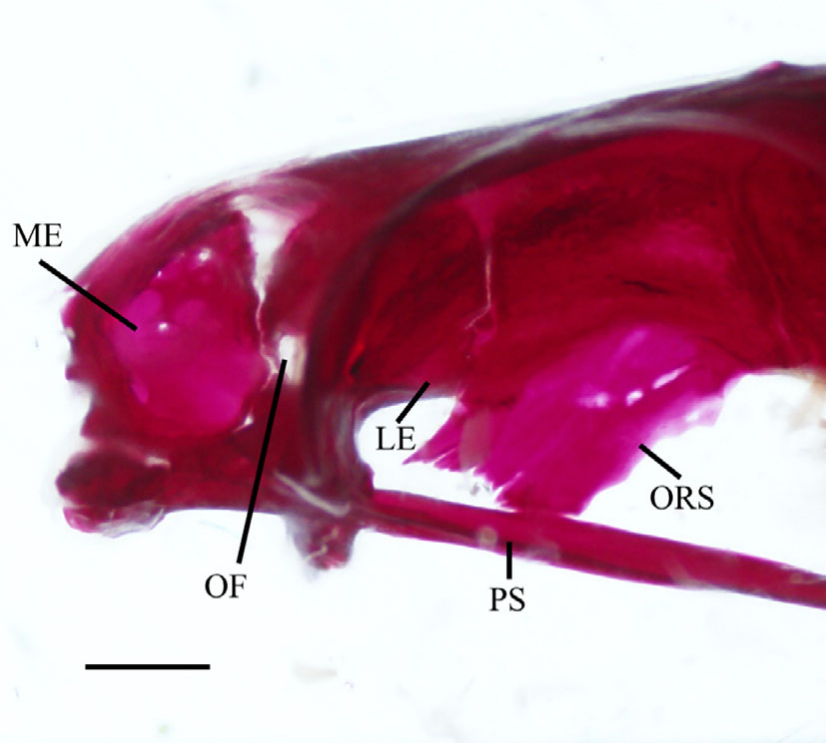
The ethmoid and orbital regions of *Hudsonius hudsonius* MZF 2507, specimen 2, in lateral view, anterior to left. Specimen shows olfactory foramen encompassed only within the lateral ethmoids and fusion of the orbitosphenoid to the parasphenoid. LE, lateral ethmoid; ME, mesethmoid portion of ethmoid block; OF, olfactory foramen; ORS, orbitosphenoid; PS, parasphenoid. Scale bar = 2 mm.

The lateral ethmoids (LE) rim the anterior region of the orbit and form the posterior portion of the nasal cavity. They articulate with the ethmoid block anteriorly, the frontals (FR) dorsally, the orbitosphenoid (ORS) posteriorly and the vomer and parasphenoid (PS) ventrally. In ventral view (2), they have a thick median margin that narrows into a set of broad, flat, lateral wings. The ventral surface is concave anteriorly, and the posterior margin forms a ridge. When viewed laterally, the lateral ethmoids narrow towards the ventral region (3). The ventral part of the anterior margin of the lateral ethmoid forms an anteriorly directed shelf, whereas the anterodorsal corner is separated from the mesethmoid portion of the ethmoid complex by cartilage. The lateral ethmoids form the posterior margin of the olfactory foramen (OF), whereas the nasal septum of the ethmoid block forms the anterior margin. In four specimens examined, the olfactory foramen was encompassed entirely within the lateral ethmoids (Figure 2). The orbitonasal foramen is observed just below the olfactory foramen, along its posterior margin.

The preethmoids sit between the vomer and ethmoid complex. They are round, ossified nodules surrounded by cartilage and are directed laterally. The vomer is a flat dermal bone located ventral to the parasphenoid and ethmoid complex. It is broadest anteriorly with round lateral edges and a single, pointed posterior process that sits between the lateral ethmoids. Its anterior end has a deep, round notch. The nasals are tubular bones that lie lateral to the supraethmoid portion of the ethmoid block. They each have three pores (Figure 1).

### 3.2 Orbital region

**FIGURE 3:**
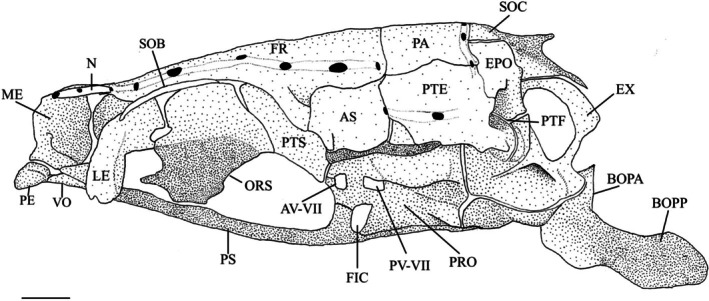
Illustration of the skull of *Hudsonius hudsonius* UAMZ F1258, specimen 1, in left lateral view, anterior to left. AV‐VII, anterior opening of the trigeminal‐facial chamber; AS, autosphenotic; BOC, basioccipital; BOPP, pharyngeal process of the basioccipital; EPO, epiotic; EX, exoccipital; FR, frontal; PV‐VII, posterior opening of the trigeminal‐facial chamber; FIC, foramen of internal carotid artery; ME, mesethmoid portion of the ethmoid block; LE, lateral ethmoids; N, nasal; ORS, orbitosphenoid; PA, parietal; PE, preethmoid; PRO, prootic; PS, parasphenoid; PTE, pterotic, PTF, posttemporal fossa; PTS, pterosphenoid; SOB, supraorbital; SOC, supraoccipital; VO, vomer. Scale bar = 1 mm.

The orbitosphenoid is a single median bone that articulates with the frontals dorsally, the lateral ethmoids anteriorly and the pterosphenoids (PTS) posteriorly (Figure 3). Its dorsal margin is divided into left and right lamellae that join in a single median ridge extending ventrally to the parasphenoid. The anteroventral corner of the medial ridge rests on a cartilage extension from the lateral ethmoids. In three specimens, the posteroventral corner is fused with the parasphenoid (Figure 3). The posterior edge of the orbitosphenoid forms an oval foramen with the lateral ethmoid. This foramen was absent in three of the specimens examined. The orbitosphenoid forms the anterior margin of the optic foramen.

**FIGURE 4:**
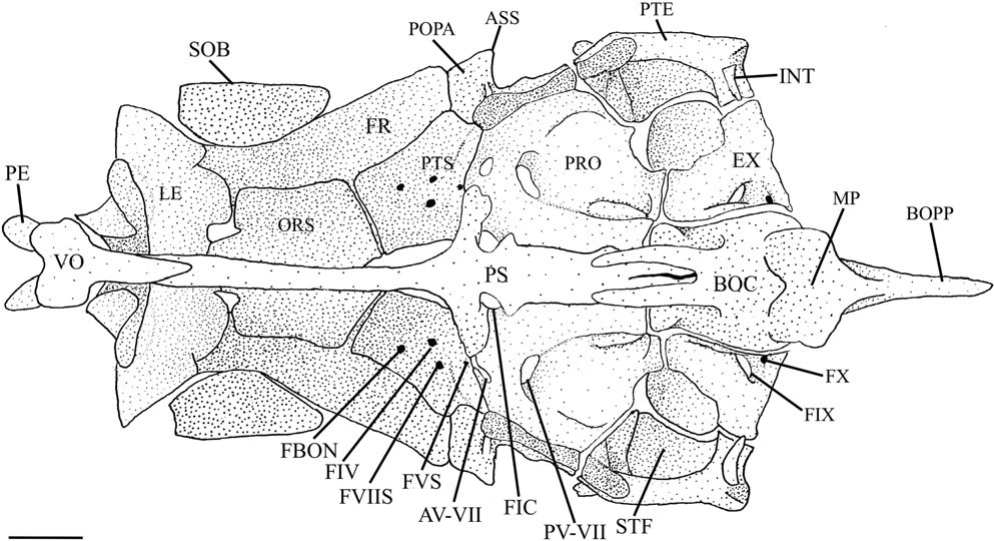
Illustration of the skull of *Hudsonius hudsonius* UAMZ F1258, specimen 1, in ventral view, anterior to top of the page. Scale bar = 1 mm. AV‐VII, anterior opening of the trigeminal‐facial chamber; ASS, autosphenotic spine; BOC, basioccipital; BOPP, pharyngeal process of basioccipital; EX, exoccipital; FIV, foramen for the trochlear nerve; FVS, foramen for ophthalmic branch of trigeminal nerve; FVIIS, foramen of superficial branch of facial nerve; FIX, glossopharyngeal foramen; FX, vagal foramen; FBON, foramen for branch of orbitonasal vein; FIC, foramen of internal carotid artery; FR, frontal; INT, intercalar; LE, lateral ethmoid; ORS, orbitosphenoid; PV‐VII, posterior opening of the trigeminal‐facial chamber; PE, preethmoid; POPA, postorbital process of the autosphenotic; PRO, prootic; PS, parasphenoid; PTS, pterosphenoid; PTE, pterotic; SOB, supraorbital; STF, subtemporal fossa; VO, vomer. Scale bar = 1 mm.

The pterosphenoid is a ventrally concave endochondral element that makes up the posterior region of the orbit (Figure 3). It articulates with the orbitosphenoid anteriorly, the frontal and autosphenotic (AS) dorsolaterally and the prootic (PRO) posteriorly. The pterosphenoids do not articulate with one another medially but instead form the posterior margins of the optic foramen and the anterior portion of the hypophyseal foramen. The posteroventral corner of the pterosphenoid forms the front edge of the anterior hyomandibular fossa. In *H. hudsonius*, there are four foramina in the pterosphenoid, accommodating a branch of the orbitonasal vein (FBON), the trochlear nerve (FIV), the superficial ophthalmic branch of the facial nerve (FVIIS) and the ophthalmic branch of the trigeminal nerve (FVS) (Figure 4). The identification of these four foramina is tentatively based on Coburn (1982).

**FIGURE 5:**
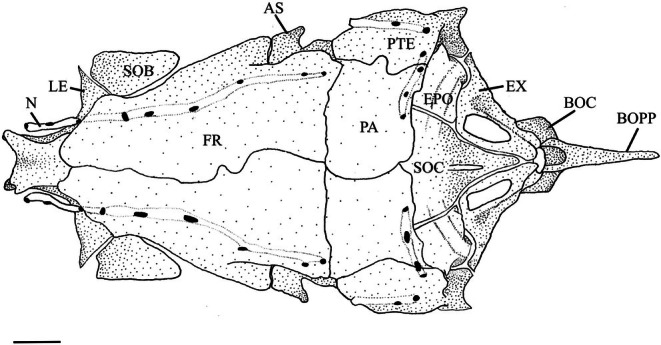
Illustration of the skull of *Hudsonius hudsonius* UAMZ F1258, specimen 1, in dorsal view, anterior to top of the page. AS, autosphenotic; BOC, basioccipital; BOPP, pharyngeal process of the basioccipital; EX, exoccipital; EPO, epiotic; FR, frontal; LE, lateral ethmoids; N, nasals; PA, parietal; PTE, pterotic; SE, supraethmoid portion of ethmoid block; SOB, supraorbital; SOC, supraoccipital. Scale bar = 1 mm.

The frontal is the largest bone of the cranium, either overlapping or underlapping its opposite medially (Figure 5). It overlaps the supraethmoid portion of the ethmoid block, supraorbital and lateral ethmoids anteriorly (Figure 5) and the orbitosphenoids and pterosphenoids posteriorly (Figure 4). Its posterolateral margin articulates with the autosphenotic, and the posterior margin overlaps the parietals (PA). When viewed dorsally (Figure 5), it narrows anteriorly, with the widest point being where the frontal contacts the autosphenotic. The frontal has a ventrally placed, laterally projecting orbital shelf that lies over and follows the curve of the orbit, narrowing anteriorly. The orbital shelf articulates posteriorly with the postorbital process of the autosphenotic (POPA) (Figure 4).

### 3.3 Otic and occipital regions

**FIGURE 6:**
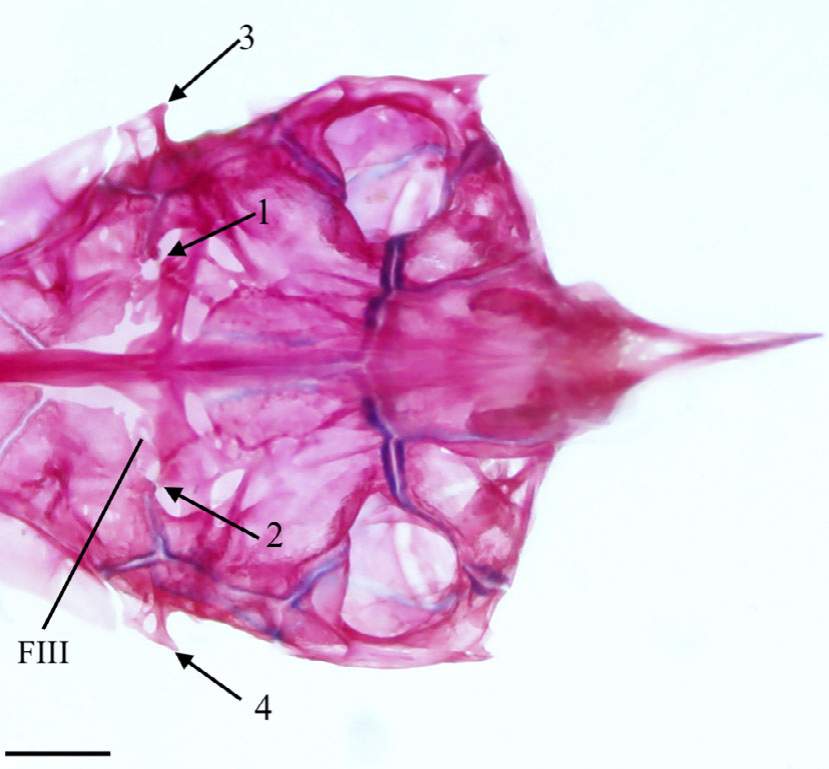
The otic, occipital and basicranial regions of the skull of *Hudsonius hudsonius* UAMZ 209, specimen 2, in ventral view, anterior to top of the page. Specimen shows bony struts that are formed over the anterior openings of the trigeminal‐facial chamber (indicated by arrows 1 and 2) and pointed, elongated, autosphenotic spines (indicated by arrows 3 and 4). FIII, oculomotor foramen. Scale bar = 1 mm.

The autosphenotic is a paired endochondral bone. It articulates with the pterosphenoid anteriorly, the frontal dorsally, the parietal posterodorsally, the pterotic (PTE) posteriorly and the prootic ventrally. The ventral border of the autosphenotic makes up half of the anterior hyomandibular facet and a portion of the posterior facet. Dorsolateral to the anterior facet is an obliquely oriented postorbital process. The posterior tip of this process is drawn out into a blunt, short and posteriorly curved autosphenotic spine (ASS) (Figure 4). In one specimen, the autosphenotic spine is sharp and elongate (Figure 6).

The prootic is a large endochondral bone with a hexagonal shape. It articulates with the pterosphenoid anteriorly and the basioccipital (BOC) posteriorly (Figure 4). It contacts the autosphenotic and pterotic, contributing to the anterior and posterior hyomandibular facets. It forms the anterior wall of the subtemporal fossa (STF), articulating with the epiotic (EPO) and exoccipital (EX) within the fossa (Figure 4). The prootic splits into two lamellae along the medial margin. The dorsal lamella meets its opposite medially and diverges anteriorly to form the posterior portion of the hypophyseal foramen. The ventral lamella forms part of the wall of a posterior myodome, with the medial margins articulating with the parasphenoid. The posterior portion is enlarged into a capsule. The anterior opening of the trigeminal‐facial chamber (AV‐VII) is usually bordered by the prootic posteriorly and the pterosphenoid anteriorly; however, this opening is fully encompassed within the prootic in one specimen. In three specimens, the anterior opening of the trigeminal‐facial chamber is fully encompassed within the prootic on one side and shared with the pterotic on the other (Figure 4). In seven specimens, I also observed a bony strut that forms over the trigeminal foramen, just lateral to the wings of the parasphenoid, on at least one side (Figure 6). The posterior opening of the trigeminal‐facial chamber (PV‐VII) is always encompassed within the prootic.

The oculomotor foramen (FIII) is found in the anteromedial corner of the prootic (Figure 6). Of the specimens whose occipital region I was able to examine, the oculomotor foramen was most commonly bordered by both the prootic and pterosphenoid. In five specimens, the oculomotor foramen was fully encompassed within the prootic on one side and shared with the pterotic on the other. In another five specimens, the oculomotor foramen was encompassed within the prootics only (as seen in Figure 6).

**FIGURE 7:**
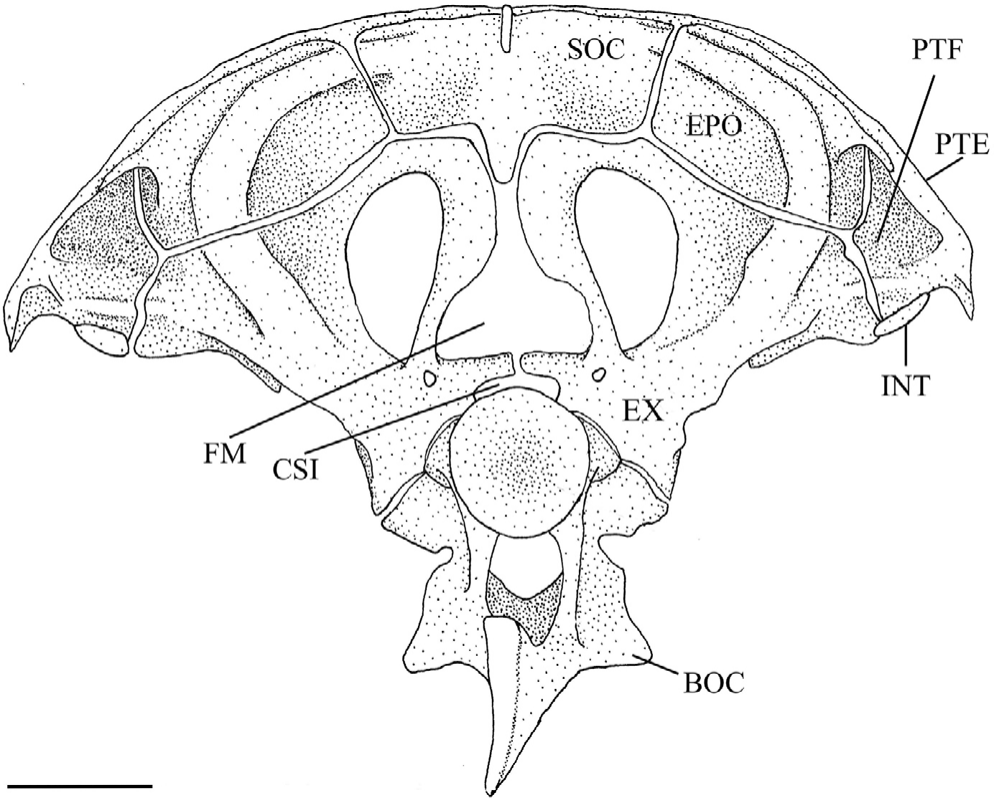
Illustration of the skull of *Hudsonius hudsonius* UAMZ 4950, specimen 1, in posterior view, dorsal to top of the page. BOC, basioccipital; CSI, cavum sinus impar; EPO, epiotic; EX, exoccipital; FM, foramen magnum; INT, intercalar; PTE, pterotic; PTF, posttemporal fossa; SOC, supraoccipital. Scale bar = 1 mm.

The pterotic is a large element that overlaps the autosphenotic anteriorly and is overlapped by the parietal dorsally (Figure 5). It forms the lateral wall of the subtemporal fossa (Figure 4), articulating with the epiotic and exoccipital within the fossa. Ventrolaterally, the pterotic forms the posterior hyomandibular facet. A short spine protrudes from the posterolateral margin of the pterotic, which is joined ligamentously to the posttemporal (PTT) and supracleithrum (SCL). In posterior view, it forms the lateral wall of the posttemporal fossa (PTF) (Figure 7).

The parietal is a rectangular dermal bone on the dorsal surface of the skull (Figure 5). It overlaps the supraoccipital (SOC) and epiotic posteriorly, the autosphenotic and pterotic laterally and is overlapped by the frontal anteriorly.

The exoccipital is a large endochondral bone that articulates with the basioccipital medially, pterotic laterally, epiotic dorsolaterally, supraoccipital dorsally and prootic anteriorly (Figures 4 and 7). In ventral view, it forms the medial portion of the subtemporal fossa (Figure 4). In posterior view, it forms the ventromedial wall of the posttemporal fossa (Figure 7). The most prominent feature of the exoccipital is a bony ring that arches posteriorly and encloses a large lateral occipital foramen. The ring has a pointed process on its posterior margin directed towards the notch formed in the second supraneural (SN2) of the second Weberian vertebra. This ring converges with its opposite posteromedially, almost touching to form a round, roughly triangular foramen magnum (FM). The exoccipitals join medially to form the floor of the foramen magnum and the roof of the cavum sinus impar (CSI) (Figure 7). In posterior view, the exoccipital forms the base of a bulge formed by the semicircular canals that continue to the epiotic and terminate along the lateral margin of the supraoccipital (Figure 7). In ventral view, the vagal (FX) and glossopharyngeal (FIX) foramina are completely encompassed within the posteromedial corner of the exoccipital. The vagal foramen lies posterior to, and is much smaller than, the glossopharyngeal foramen (Figure 4).

The epiotic is a paired endochondral bone that articulates with the supraoccipital medially, exoccipital ventrally and parietal dorsally (Figure 7). It contribute to the dorsal portion of the subtemporal fossa. In posterior view, it forms the dorsomedial wall of the posttemporal fossa and the floor of the cavum sinus impar (Figure 7). It has a prominent bulge formed by the semicircular canal along its posterior surface (Figure 7). A laterally facing shelf is formed along the dorsolateral margin of this bulge for the posttemporal to articulate and form the roof of the posttemporal fossa (Figure 7). A weak tubercle is also observed on the medial margin of the bulge.

The supraoccipital is a median endochondral bone that articulates epiotics laterally and exoccipitals ventrally (Figure 7), whereas the parietals overlap its anterodorsal margin (Figure 5). In dorsal view, the supraoccipital has a triangular shape and is anteriorly convex and dorsomedially concave with a small sagittal crest. The supraoccipital forms the dorsal margin of the foramen magnum. Its posterolateral surface has a bulge formed by the semicircular canal on either side that terminates before the sagittal crest.

The intercalars (INT) are paired dermal ossifications. They are crescentic and lie along the posterior margin of the pterotic, close to the pterotic‐exoccipital suture (Figures 4 and 7). They are small in *H. hudsonius*.

### 3.4 Basicranial region

The basioccipital is a single posteroventrally positioned element (Figures 5 and 7). It articulates with the exoccipitals dorsally, prootics anteriorly and parasphenoid ventrally (Figures 4 and 7). Posteriorly, it articulates with the first vertebral centrum. The pharyngeal process (BOPP) is found ventrally and is directed posteriorly (Figure 3). It is laterally compressed, widens posteriorly and had an almost rectangular shape with a round posterior end that does not quite reach the os suspensorium (OS). Anterior to the pharyngeal process is a concave, anteroventrally facing masticatory plate with squared, ventrolaterally projecting wings, a narrow posterior margin and a notched anterior margin (Figure 4). Dorsal to the plate are supporting arches that connect the pharyngeal process and plate to the ventrolateral portion of the basioccipital (Figure 7). A large canal is formed between the arches. The anteroventral margin of the basioccipital is notched, forming a portion of the walls and roof of the posterior myodome. Two ventral lamellae extend over the anterior edge of the basioccipital and dorsal margins of the prootics (Figure 4). These lamellae overlap the posterior end of the parasphenoid to create a ventral floor that helps to close the posterior myodome along with the parasphenoid. In one of the specimens examined, the ventral lamellae are absent, but this closure of the posterior myodome is still observed.

The parasphenoid is a single bone and the longest of the cranium. It can be divided into three parts. First is the anterior shaft, which passes between the left and right orbits (Figure 3) and terminates dorsal to the vomer (Figure 4). Second, there are two dorsally directed ascending wings that suture to the anteromedial margins of the prootics and form the anterior margin of the foramen for the internal carotid artery (FIC). Third is the forked posterior end. The medial margins of the fork abut and create a portion of the roof of the closed posterior myodome (CPM) (Figure 4).

### 3.5 Branchial apparatus

**FIGURE 8:**
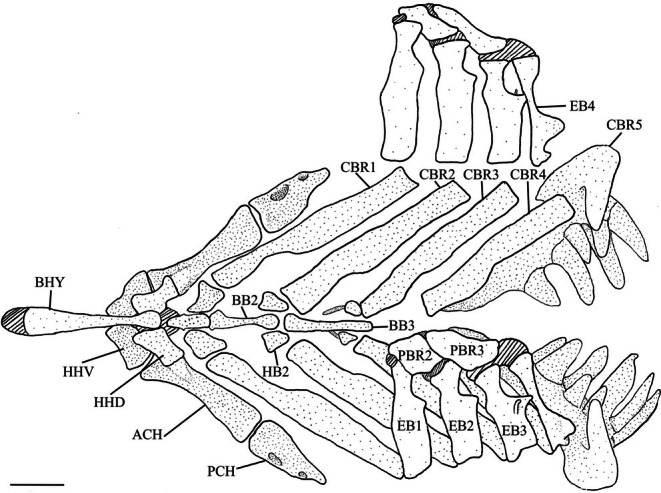
Illustration of the branchial apparatus and hyoid bars of *Hudsonius hudsonius* UAMZ 4950, specimen 1, in dorsal view, anterior to left. Upper elements on the right side are reflected away and are in ventral view. Hatching represents cartilage. ACH, anterior ceratohyal; BB, basibranchial; BHY, basihyal; CBR, ceratobranchial; EB, epibranchial; HB, hypobranchial; HHD, dorsal hypohyal; HHV, ventral hypohyal; PBR, pharyngobranchial; PCH, posterior ceratohyal. Scale bar = 1 mm.

**FIGURE 9:**
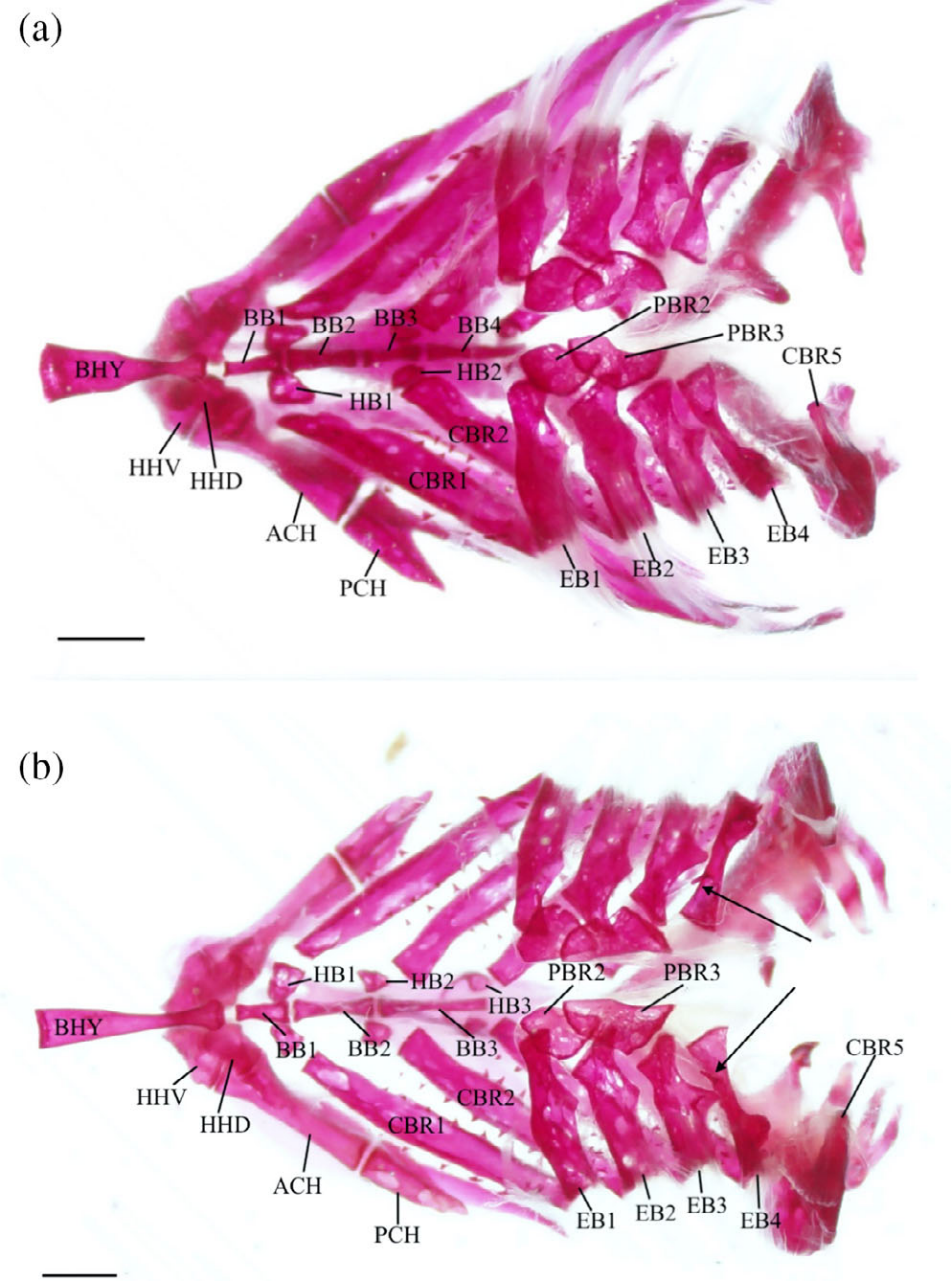
The branchial apparatus of *Hudsonius hudsonius* MZF 680, specimen 3 (a), MZF 2506, specimen 1 (b), in dorsal view, anterior to left. Specimen (a) shows four epibranchials instead of three. Specimen (b) shows dorsal processes on the fourth epibranchials (indicated by arrows). ACH, anterior ceratohyal; BB, basibranchial; BHY, basihyal; CBR, ceratobranchial; EB, epibranchial; HB, hypobranchial; HHD, dorsal hypohyal; HHV, ventral hypohyal; PBR, pharyngobranchial; PCH, posterior ceratohyal. Scale bars = 1 mm.

The pharyngobranchials (PCH) are the dorsalmost elements of the branchial apparatus (Figure 8); there are only two pairs of pharyngobranchials, representing pharyngobranchials 2 and 3. These are small bones with irregular edges. The second pharyngobranchial (PBR2) has a roughly subcircular shape and is joined by cartilage to the first epibranchial (EB1) along its anterolateral margin and the second epibranchial along the posteromedial margin. The third pharyngobranchial is more elongate than the second, overlying a quarter of the posterodorsal end of the second pharyngobranchial, and is of similar width. Its median edge is sigmoidal, whereas the lateral edge is semicircular. In four of the branchial apparatuses examined, the medial edge has a deep notch (Figure 9a). The second and third pharyngobranchials are connected medially by cartilage. The posterolateral edge of the third pharyngobranchial is joined by cartilage to the third epibranchial, whereas its posterior edge joins to the fourth epibranchial (EB4), also by cartilage.

Four pairs of epibranchials are present, attaching to the ceratobranchials (CBR) at their distal ends. All the epibranchials are slightly concave dorsally and have a shallow notch and a posteriorly projecting uncinate process along the posterior margin. The first three epibranchials are constricted towards their dorsal ends next to the uncinate process. The first epibranchial is narrower than the second or third, and its anterior margin is gently convex. Its posterior notch and uncinate process are weakly developed. The second epibranchial has a straight anterior margin, with a better‐developed posterior notch and uncinate process. The third epibranchial is shorter than the first and second epibranchials. Its distal end is wider than its proximal end, with a pronounced posterior notch and a long, slender, uncinate process that overlaps the fourth epibranchial. A thin, broad process is found on the dorsal surface of the third epibranchial, originating laterally to the uncinate process. In four of the branchial apparatuses examined, this dorsal process is present on only one side. The fourth epibranchial is slenderer than the others. Its distal end is narrower, and it is constricted in the middle, unlike the other epibranchials (Figure 8). A thin, medially directed dorsal process of varied size and position can sometimes be found in close proximity to the uncinate process of the third epibranchial (Figure 9b). This dorsal process was seen on only one epibranchial in 11 of the examined specimens and was either present on both or entirely absent in 6. The uncinate process of the fourth epibranchial is broad, flat and round, positioned closer to the distal end, and directed posterodorsally.

The ceratobranchials are the largest elements of the gill arches. The first four are ventrally concave, narrow and elongate, contacting their respective epibranchials at their distal ends. The first ceratobranchial (CBR1) is the longest, whereas the second (CBR2), third (CBR3) and fourth (CBR4) ceratobranchials get progressively shorter. The proximal end is narrower than the distal end for the first and third ceratobranchials and wider for the second ceratobranchial. Both ends are equal for the fourth ceratobranchial. The proximal portion of the first ceratobranchial is slightly constricted for about one‐third of the bone before widening distally.

**FIGURE 10:**
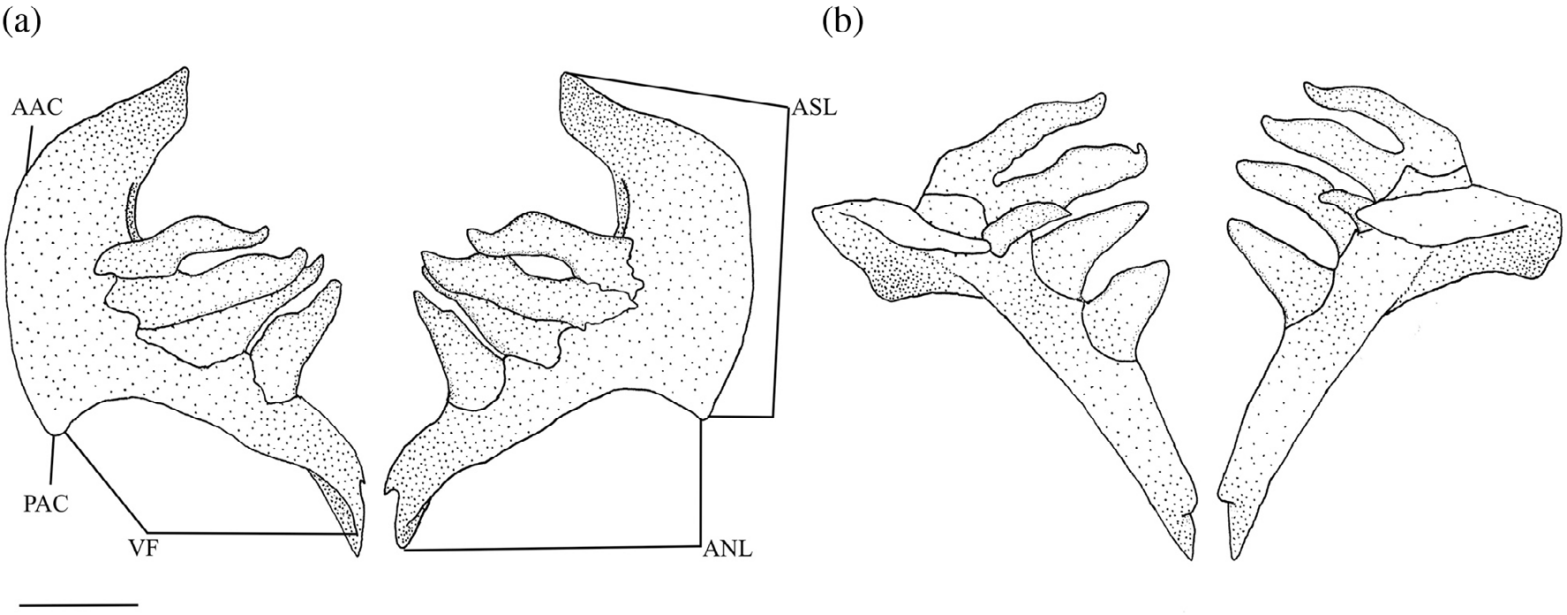
Illustration of the pharyngeal arches of *Hudsonius hudsonius* UAMZ F1258, specimen 1, in (a) posteroventral view, dorsal to top of the page and (b) dorsal view, anterior to bottom of the page. AAC, anterior angle of fifth ceratobranchial; ANL, anterior limb of fifth ceratobranchial; ASL, ascending limb of fifth ceratobranchial; VF, ventral flange of fifth ceratobranchial. Scale bar = 1 mm.

**FIGURE 11:**
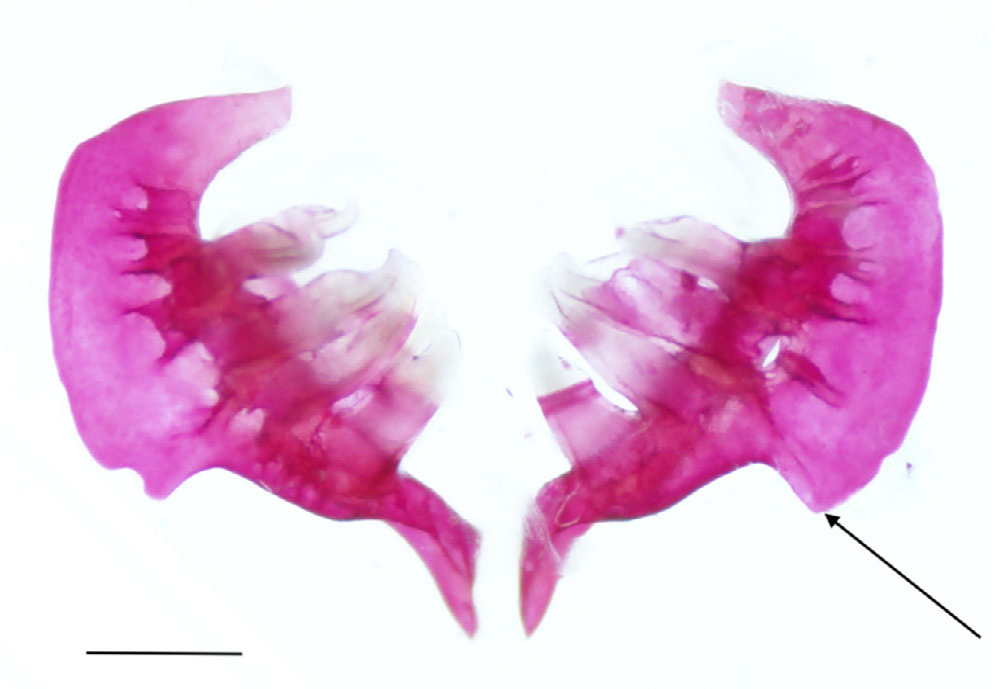
The pharyngeal arches of *Hudsonius hudsonius* UAMZ 4950, specimen 1, in posteroventral view, dorsal to top of the page. Specimen shows broadened posterior angle (indicated by arrow). Scale bar = 1 mm.

The pharyngeal teeth are supported on the fifth ceratobranchial (Figure 10). This bone can be divided into two limbs: the anterior (ventral) limb and the ascending (posterior) limb. The anterior limb (ANL) runs parallel to the other ceratobranchials but is more ventrally positioned. When viewed laterally, the anterior limb narrows anteriorly into a blunt point with a ventral flange (VF) that runs along the lateral margin and tapers ventrally. The ascending limb (ASL) is broader and curves dorsally. The two limbs meet in an acute, blunt anterior angle (AAC). The ascending limb is weakly arched, with a weak, undeveloped posterior angle. In one set of pharyngeal arches examined, the anterior angle of the ascending limb was broad and round (Figure 11).

The pharyngeal teeth are long and conical with hooked ends. The majority of specimens had a dental formula of 1.4–4.1 (Table 3), which was counted on 25 of the sets of ceratobranchial arches. The most common dental formula that deviated from 1.4–4.1 was 2.4–4.2 (five sets) followed by 0.4–4.0 and 2.4–4.1 (both four sets) and 1.4–4.0 (three sets). The formulae 1.4–4.2 and 0.3–4.0 were observed only once each. In one of the sets examined, there appeared to be three rows of teeth instead of two on the left arch. However, this is likely due to a failure of some functional teeth, in this case, the first and fourth teeth on the major row, to fall out when its replacement ankylosed with the arch (Evans & Deubler, 1955).

Three pairs of nodular, irregularly shaped bones, the hypobranchials (HB), are located between the first three ceratobranchials and basibranchials (BB). The first pair is the largest, followed by the second and third. The first (HB1) and second hypobranchial (HB2) pairs have short, blunt ventral processes. The third pair of hypobranchials (HB3) has a set of long, slender ventral processes that hook out anteriorly.

The three basibranchials are unpaired bones found along the midline of the branchial apparatus. The first basibranchial (BB1) is the shortest and tapers posteriorly in dorsal view. In lateral view, it has a triangular shape and a constricted anterior end. A notch on either side at the midsection of the bone accommodates the articulation of the first pair of hypobranchials. The second (BB2) and third (BB3) basibranchials are rod‐shaped. The second basibranchial is constricted in the middle, and the anterior and posterior ends are equal in width. The third basibranchial is the longest and slender, tapering posteriorly.

The basihyal (BHY) is a long, unpaired bone and the anteriormost element of the branchial apparatus. It curves up dorsally and has an anterior cartilaginous tip. The deepest point of the curve is towards the anterior end, about one‐third of the way down, with a ventral ridge. The posterior end of the basihyal articulates with the first basibranchial and has two tubercles on either side for ligaments to anchor it to the ventral hypohyals (HHV). The shaft of the basihyal narrows posteriorly. The posterior end is bulbous and wider than the narrowest point of the bone but still narrower than the anterior portion.

In one of the branchial apparatuses I examined (MZF 680), there are four basibranchials instead of three (Figure 9a). In this specimen, the fourth, posteriormost basibranchial is the longest, followed by the second, third and first. The hypobranchials lie between the first and second, third and fourth basibranchials and at the end of the fourth basibranchial. The first basibranchial is essentially the same as described above. The second basibranchial is widest at the anterior end, tapering posteriorly. The anterior and posterior ends of the third basibranchial are equal, and the middle is constricted. The fourth basibranchial is uniform in overall shape, and the anterior end is slightly wider.

### 3.6 Hyoid region

**FIGURE 12:**
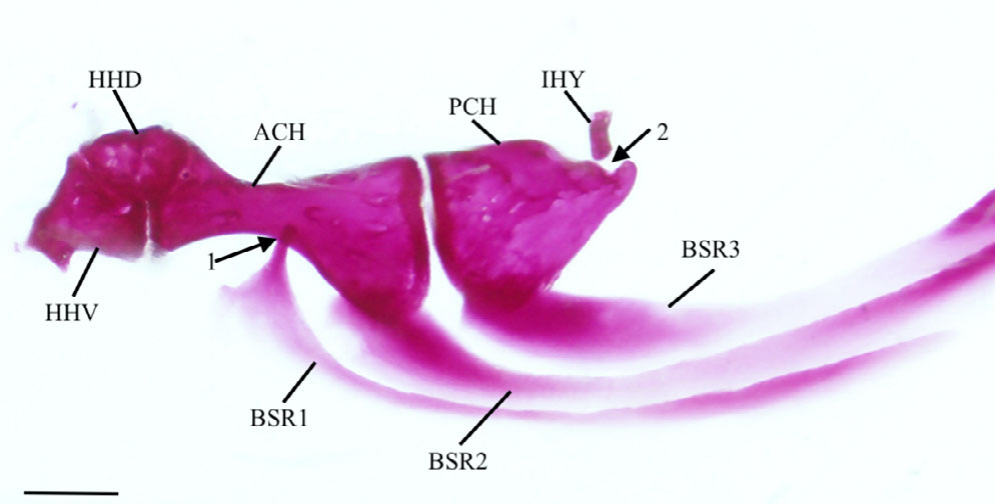
The hyoid bar or *Hudsonius hudsonius* UAMZ F9126, specimen 2, in medial view, anterior to left (image reversed). Specimen shows an elongate head on the first branchiostegal ray (indicated by arrow 1) and a deepened posterior notch on the posterior ceratohyal (indicated by arrow 2). ACH, anterior ceratohyal; BSR, branchiostegal ray; HHD, dorsal hypohyal; HHV, ventral hypohyal; IHY, interhyal; PCH, posterior ceratohyal. Scale bar = 0.5 mm.

The interhyal (IHY) is a small, cylindrical bone that attaches to the medial surface of the hyomandibula‐symplectic junction with cartilage at one end, with the other articulating in a divot found on the posterodorsal margin of the posterior ceratohyal (PCH) (Figure 12).

**FIGURE 13:**
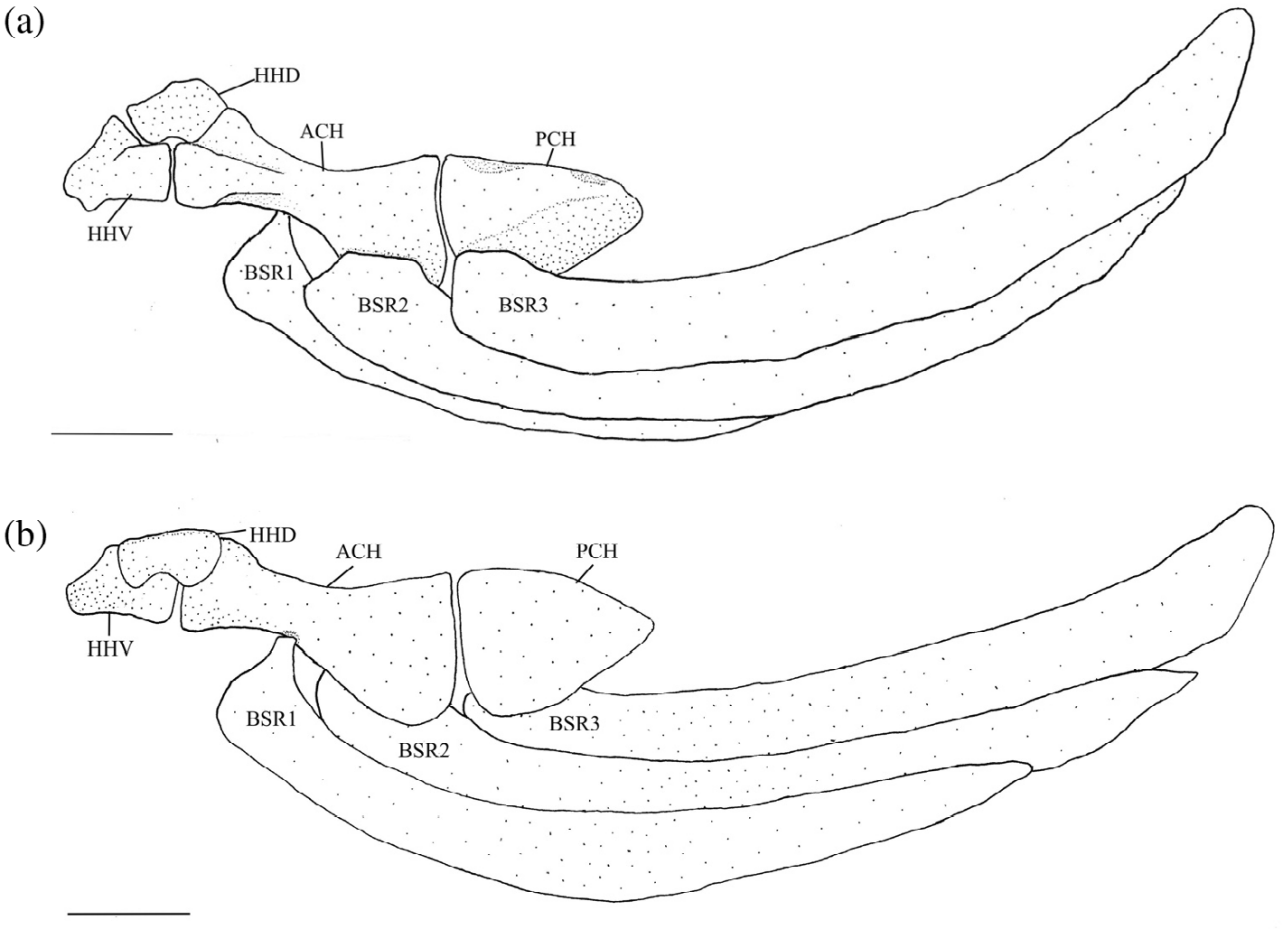
Illustration of the left hyoid bar of *Hudsonius hudsonius* UAMZ 4950, specimen 1, in (a) lateral and (b) medial view, anterior to left (image b reversed). ACH, anterior ceratohyal; BSR, branchiostegal ray; HHD, dorsal hypohyal; HHV, ventral hypohyal; PCH, posterior ceratohyal. Scale bars = 1 mm.

The posterior ceratohyal (Figure 13) is triangular. It has two superficial foramina on its dorsal edge that are connected under a bridge of bone. The anterior opening is larger than the posterior opening. Posterior to the foramina is a notch where the interhyal articulates. This notch is noticeably deeper in five of the specimens examined (Figure 12).

The anterior ceratohyal (ACH) is widest at its edge that contacts the posterior ceratohyal. It is tapered in the middle, then widens into a slightly twisted head at its anterior end. The anterodorsal portion articulates with the dorsal hypohyal (HHD), whereas the anteroventral portion articulates with the ventral hypohyal. A small notch is found on the medial side at the narrowest point of the anterior ceratohyal where the anteriormost branchiostegal ray (BSR) articulates.

The ventral hypohyal is larger than the dorsal hypohyal. The dorsal hypohyal is an angular bone articulating with the ventral and the anterior ceratohyal along its ventral surface. The posterior portion of the basihyal rests between the right and left dorsal hypohyals and is ligamentously attached to each element. A tubercle is observed along the dorsomedial margin of the dorsal hypohyal. The ventral hypohyal is triangular, articulating with the anterior ceratohyal and dorsal hypohyal. A small tubercle is observed on its anterolateral surface.

**FIGURE 14:**
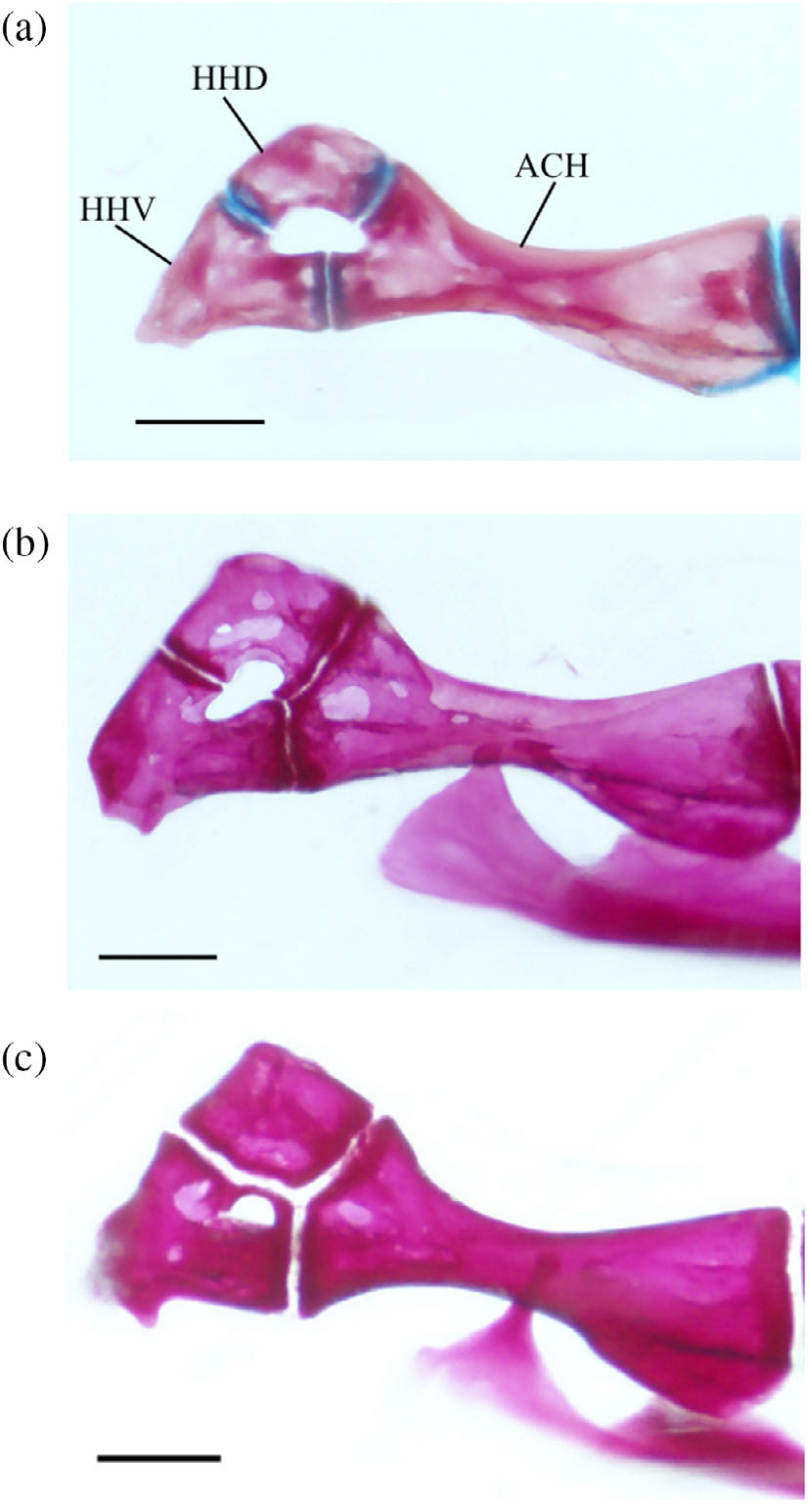
The left hypohyal foramen of *Hudsonius hudsonius* UAMZ 209, specimen 1, (a) UAMZ F9126, specimen 2, (b) UAMZ 4950, specimen 1, (c) in medial view, anterior to left (images reversed). The hypohyal foramen can be partly formed by the anterior ceratohyal and the dorsal and ventral hypohyals (a), formed by only the dorsal and ventral hypohyals (b) or only within the ventral hypohyal (c). ACH, anterior ceratohyal; HHD, dorsal hypohyal; HHV, ventral hypohyal. Scale bars = 0.5 mm.

The anterior ceratohyal and the dorsal and ventral hypohyals variably form the hypohyal foramen (Figure 14). In 26 of the hyoid bars examined, the hypohyal foramen is formed by all three elements (Figure 14a), giving the anterior ceratohyal a shallowly forked appearance. In 18 of the hyoid bars examined, the hypohyal foramen is encompassed within the dorsal and ventral hypohyals, but the anterior ceratohyal does not contribute (Figure 14b). In two of the hyoid bars examined, the hypohyal foramen is encompassed only within the ventral hypohyal (Figure 14c).

The three branchiostegal rays (Figure 13) are all similar in shape. The first branchiostegal (BSR1) is the smallest and slenderest, tapering anteriorly into a short, blunt head that articulates with the medial surface of the anterior ceratohyal. In 16 of the hyoid bars examined, the head is elongate (Figure 12). The base ventral to this head is expanded with a broad, round anterior margin, the clupeoid projection (Coburn, 1982). The heads of the second and third branchiostegals are much broader than the first, whereas the head of the second branchiostegal is the largest. The second branchiostegal (BSR2) articulates with the lateral side of the anterior ceratohyal, whereas the third (BSR3) articulates with the lateral side of the posterior ceratohyal. The third branchiostegal ray is the broadest.

**FIGURE 15:**
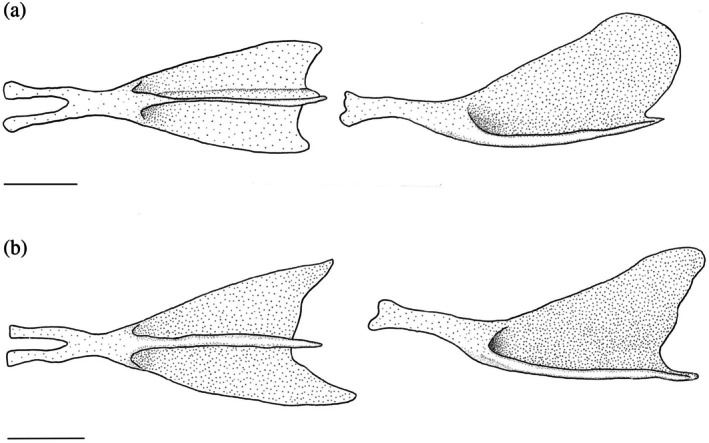
Illustration of the urohyal of *Hudsonius hudsonius* UAMZ 1525, specimen 3 (a) UAMZ 4950, specimen 1 (b) in dorsal (left) and lateral (right) view, anterior to left. Specimen (a) shows a rounded dorsal wing that is slightly longer than the ventral wings. Specimen (b) shows a triangular dorsal wing that is shorter than the ventral wings. Scale bars = 1 mm.

**FIGURE 16:**
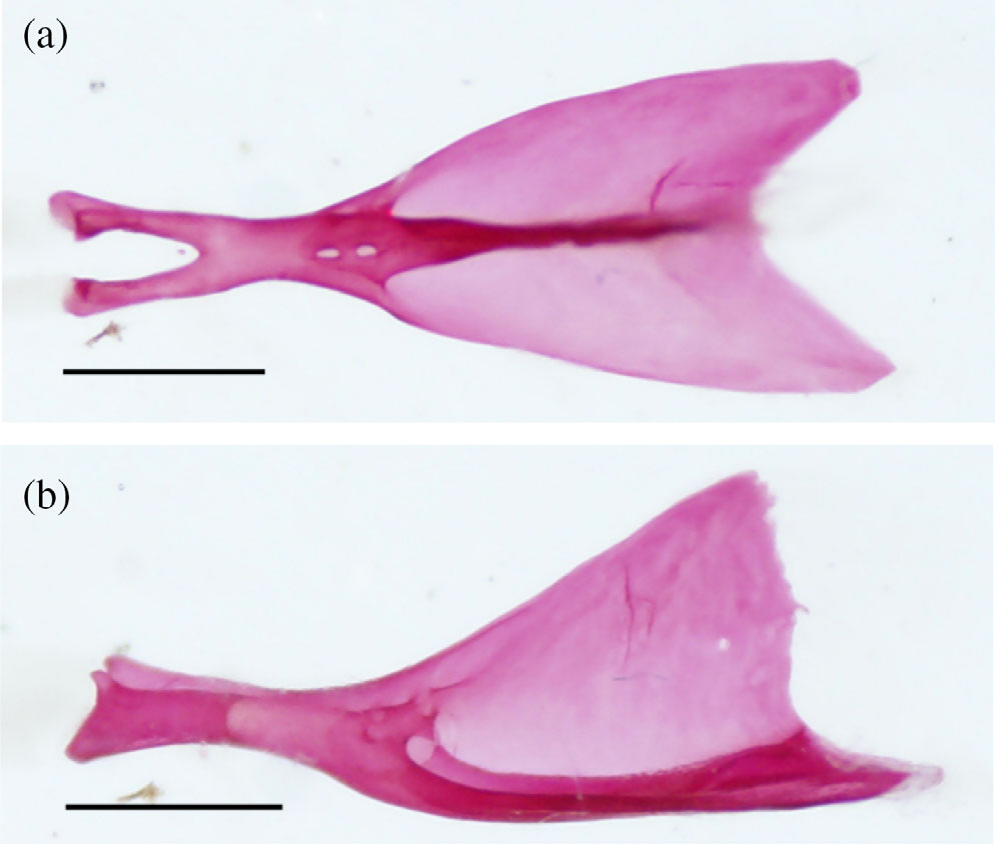
The urohyal of *Hudsonius hudsonius* MZF 2506, specimen 1, in (a) dorsal and (b) lateral view, anterior to left. Scale bar = 1 mm.

The urohyal has two horizontal ventral wings and a single vertical dorsal wing. All three wings taper together anteriorly into a constricted, cylindrical neck, bifurcating into two processes separated by a distinct notch. These processes attach to the ventral hypohyals via ligaments. In 27 of the hyoid bars examined, the dorsal wing is slightly longer than the ventral wings and has a broad, semicircular posterior margin (Figure 15a). In 19 of the hyoid bars examined, the ventral wings are longer than the dorsal wings, and the dorsal wing itself has a triangular shape with a round posterodorsal corner (Figure 15b). One urohyal has a distinct shape: the ventral wings are much longer than the dorsal wing, and the posterior margin of the dorsal wing is almost straight (Figure 16).

### 3.7 Opercular series

**FIGURE 17:**
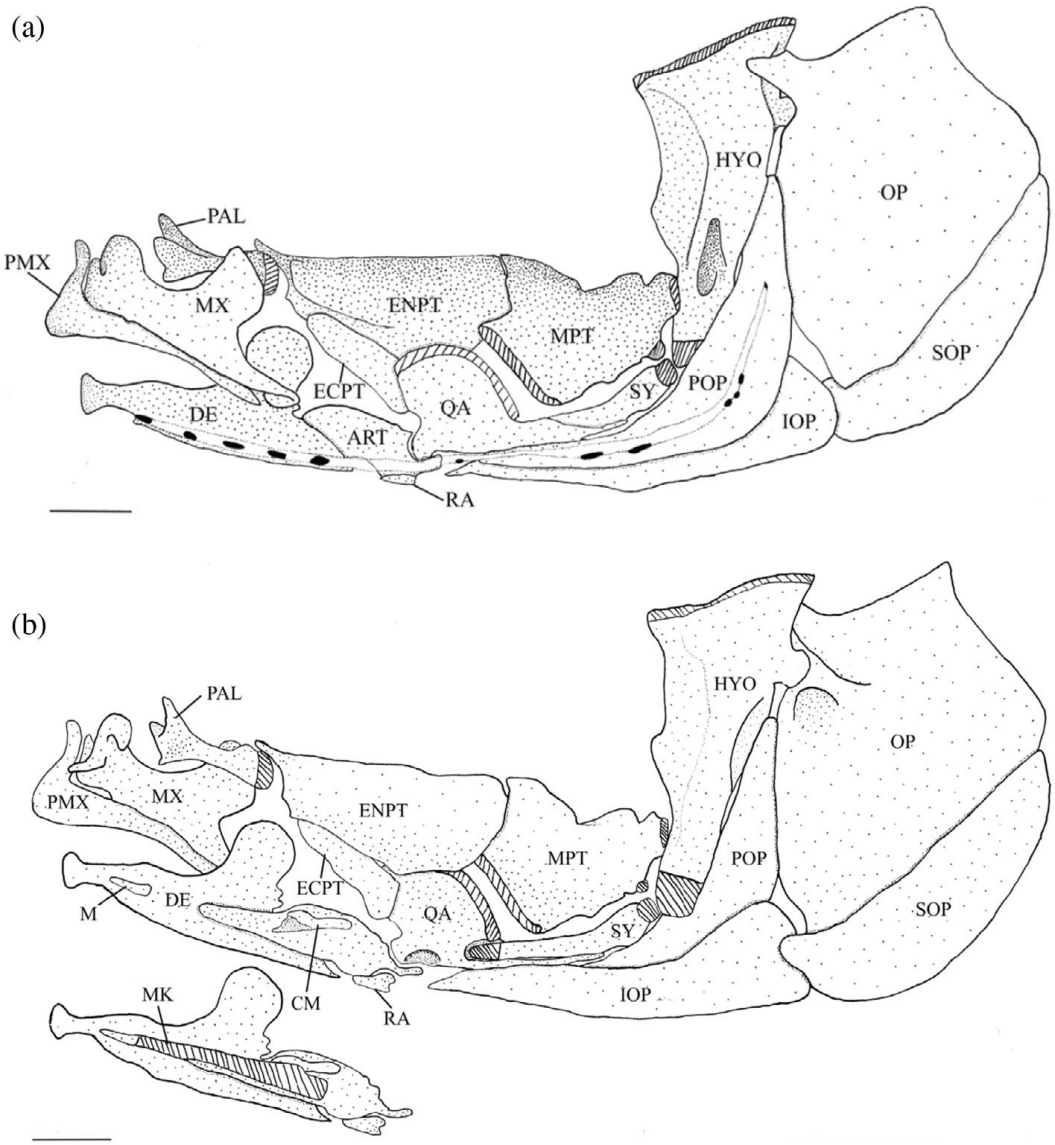
Illustration of the left opercular series, suspensorium and oral jaws of *Hudsonius hudsonius* UAMZ 3215, specimen 1, in (a) lateral view, anterior to left, and (b) medial view, anterior to left (image b reversed). Hatching represents cartilage. ART, anguloarticular; AWH, anterior wing of the hyomandibular; CM, coronomeckelian; DE, dentary; ECPT, ectopterygoid; ENPT, endopterygoid; HYO, hyomandibular; IOP, interopercle; M, mentomeckelian; MPT, metapterygoid; MX, maxilla; OP, opercle; PAL, palatine; PMX, premaxilla; POP, preopercle; QA, quadrate; RA, retroarticular; SOP, subopercle; SY, symplectic. Scale bars = 1 mm.

Four bones make up the opercular series (Figure 17). The opercle (OP) is the largest bone. It is concave medially and the anterodorsal corner is drawn into a short, blunt arm, the opercular process, that overlaps the posterior margin of the hyomandibula (HYP). The anteroventral edge of the opercle is convex and is overlapped by the preopercle (POP). The anterodorsal edge is shallowly concave and shorter than the posteroventral edge. The posterodorsal edge is also sigmoidal with a distinct posterodorsal corner. The opercular facet is found just beneath the anterodorsal process on the lateral side. It is a deep socket that joins the opercle to the hyomandibular.

The subopercle (SOP) is crescentic with a straight, anterodorsal edge, whereas the posteroventral edge is convex. The anterodorsal corner of the subopercle is drawn into a short anterodorsal process that articulates with the medial side of the interopercle (IOP). The branchiostegal rays lie along the medial side of the subopercle when they are at rest.

The interopercle is a wedge‐shaped bone with a concave dorsal margin that underlies the preopercle. The posterior margin of the interopercle is convex. The interopercle is widest at its posterior end, tapering anteriorly into a fine point that ligamentously attaches to the retroarticular (RA). Medially, it contacts the interhyal and posterior ceratohyal, overlapping dorsolateral margin of the interopercle.

The preopercle is a curved bone with distinct vertical and horizontal limbs; the vertical limb is slightly longer. The hyomandibula‐symplectic‐interhyal junction lies along the lateral margin of the vertical limb. The posterior flange of the hyomandibula overlaps the anterodorsal margin of the vertical limb. The anterodorsal margin of the horizontal limb articulates underneath the ventral groove of the quadrate (QA).

### 3.8 Suspensorium

**FIGURE 18:**
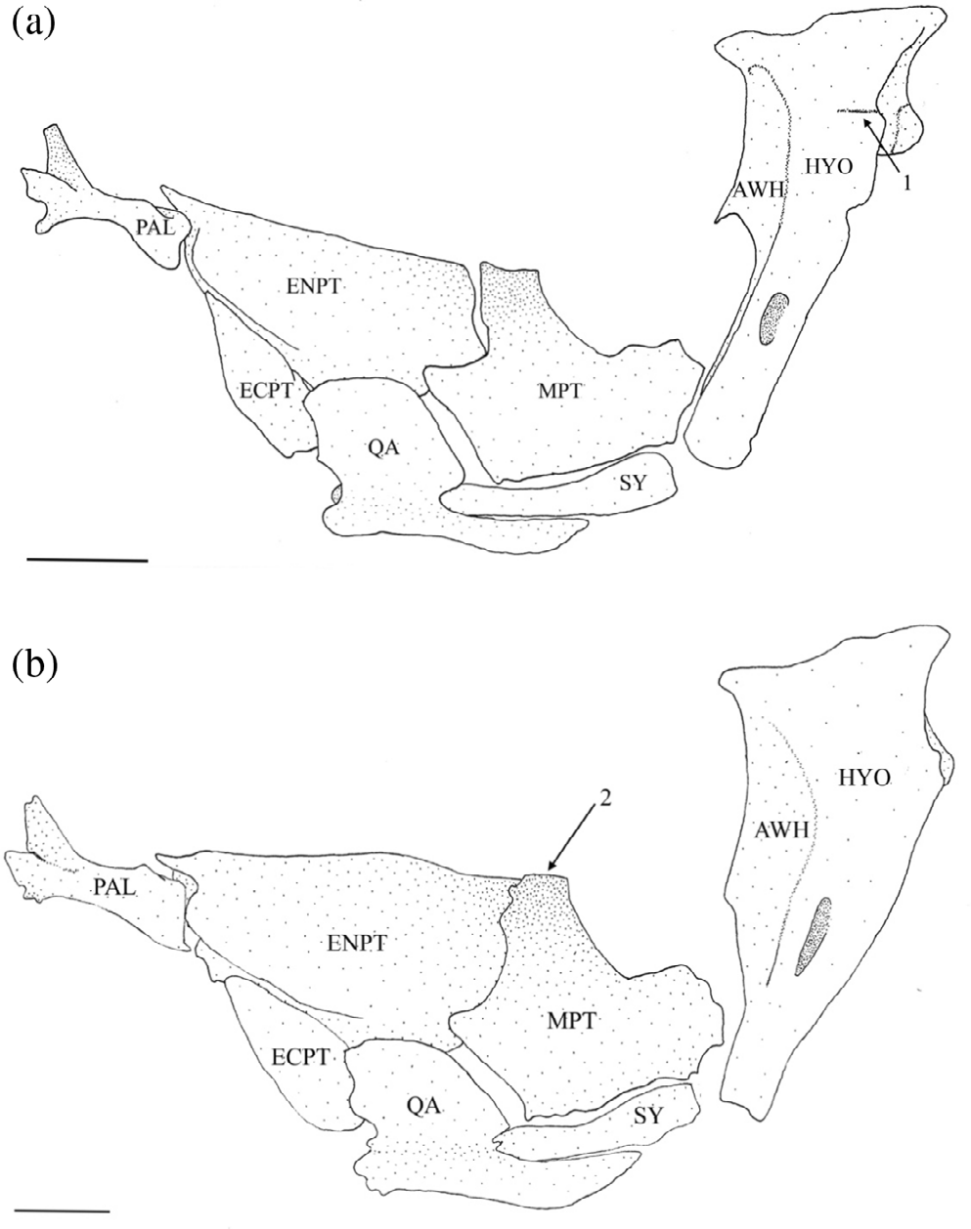
Illustration of the left suspensorium of *Hudsonius hudsonius* F1258, specimen 1 (a), MZF 2605, specimen 1 (b) in lateral view, anterior to left. Arrow 1 points to a weakly developed adductor ridge. Arrow 2 points to a broad, squared anterodorsal process of the metapterygoid. AWH, anterior wing of the hyomandibular; ECPT, ectopterygoid; ENPT, endopterygoid; HYO, hyomandibular; MPT, metapterygoid; PAL, palatine; QA, quadrate; SY, symplectic. Scale bar = 1 mm.

The hyomandibula (Figure 17) is the posteriormost element of the suspensorium. It is widest at its dorsal end, forming a head with anterior and posterior condyles, which articulate with the skull in corresponding anterior and posterior fossae formed by the autosphenotic, pterosphenoid and prootic. The hyomandibula tapers ventrally, ending in a cartilaginous cap that articulates and forms a junction with the metapterygoid (MPT), symplectic (SY) and interhyal. The anteroventral margin of the hyomandibula articulates with the metapterygoid at two points: the ventral point being part of the junction with the symplectic and interhyal, and the dorsal point articulating with the hyomandibula just ventral to the termination of the anterior wing (AWH). The anterior wing originates at the anterior margin of the head of the hyomandibula and extends roughly two‐thirds of the length of the bone. In 17 of the suspensoria examined, the anterior wing is constricted towards the ventral end and drawn into a distinct anteroventral point (Figure 18a). The posterior wing starts below the posterior margin of the head and terminates towards the ventral end of the bone, overlapping the vertical arm of the preopercle. The opercular condyle protrudes posteriorly, articulating with the opercle. In four of the specimens examined, a weakly developed adductor ridge is present at the level of the opercular condyle (Figure 18a), whereas in one specimen, the adductor ridge is developed into a distinct flange.

The symplectic (Figure 17) is a compressed, rod‐shaped, dorsally curved bone with round cartilaginous anteroventral and posterodorsal ends. The posterodorsal end is part of the hyomandibula‐symplectic‐interhyal junction. The dorsal edge of the anteroventral end inserts beneath a groove formed by the quadrate. Usually, the dorsal edge of the symplectic is separate and does not articulate with the metapterygoid. However, in 19 of the suspensoria examined, the dorsal edge of the symplectic does articulate with the metapterygoid.

**FIGURE 19:**
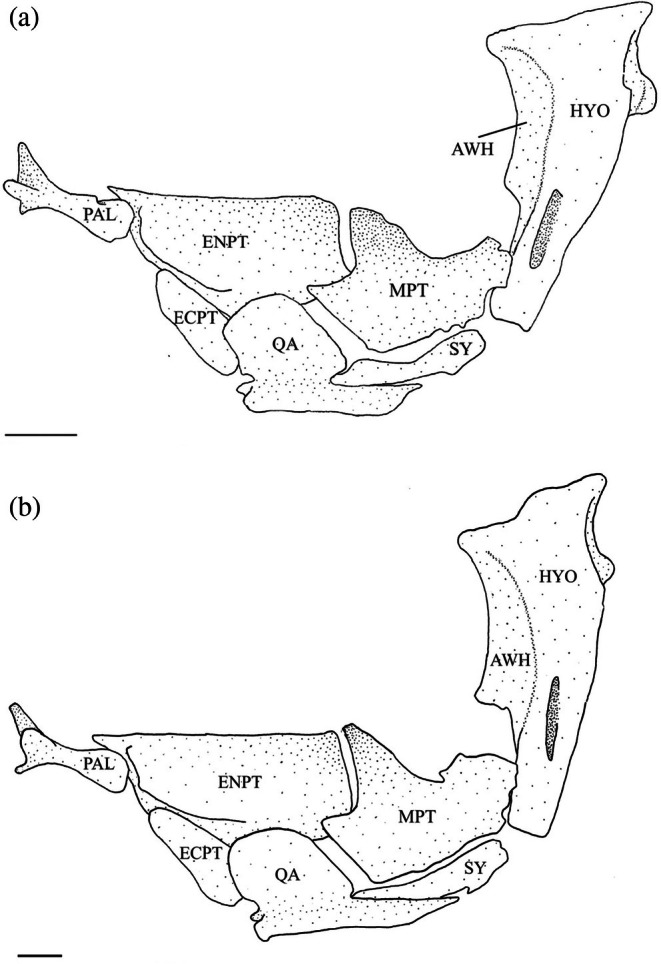
Illustration of the left suspensorium of *Hudsonius hudsonius* UAMZ 4950, specimen 1 (a), UAMZ 1525, specimen 3 (b) in lateral view, anterior to left. The metapterygoid of specimen (a) had a rounded anterodorsal process. For specimen (b), the anterodorsal process of the metapterygoid is pointed. AWH, anterior wing of the hyomandibular; ECPT, ectopterygoid; ENPT, endopterygoid; HYO, hyomandibular; MPT, metapterygoid; PAL, palatine; QA, quadrate; SY, symplectic. Scale bar = 1 mm.

The metapterygoid is a broad bone that curves dorsomedially. It typically overlaps the anterior margin of the endopterygoid (ENPT) completely (Figures 17 and 18b). In 11 of the suspensoria examined, the metapterygoid partially overlaps the endopterygoid, covering only the posteroventral corner (Figure 19a). The posterior margin of the metapterygoid has two distinct cartilaginous heads. The smaller one articulates with the junction formed by the symplectic, hyomandibula and interhyal, whereas the larger one articulates with the anterior margin of the hyomandibula. The dorsal edge is concave, whereas the anterodorsal corner is drawn into a process that is aligned with the dorsal edge of the ectopterygoid (ECPT). The anterodorsal process typically has a broad, square shape (Figure 18b). In seven of the suspensoria examined, the anterodorsal process has a round shape (Figure 19a), whereas in another seven, the anterodorsal process has a thin, pointed shape (Figure 19b). In one specimen, the anterodorsal process is indistinct (Figure 17). A weakly developed process is found at the posterodorsal corner of the metapterygoid.

The quadrate has a dorsal semicircular blade whose anterior and dorsal margins overlap the ectopterygoid and endopterygoid (Figure 17a). The anteroventral corner forms a round condyle that articulates with the anguloarticular. Ventral to the blade is a long, ventrally grooved posterior process that accommodates the symplectic and articulates with the dorsal edge of the horizontal limb of the preopercle (Figure 17b). The ventral base of the quadrate has a medially placed fossa.

The endopterygoid is larger than the metapterygoid. It curves medially. The lateral side is overlapped by the ectopterygoid anteriorly, the quadrate ventrally and the metapterygoid posteriorly (Figure 17). The anterodorsal corner of the endopterygoid has a socket that articulates with the posterior end of the palatine (PAL). Dorsal to the facet is a pointed, medially directed anterior process extending over the palatine. Another ventrally placed socket is present posterior to this process, which accommodates the posterior margin of the lateral ethmoid. In one specimen, the anterior process is reduced and rudimentary. A flange forms along the lateral margin of this socket and extends posteroventrally along the lateral side of the endopterygoid (Figure 17a). The dorsal edge of the endopterygoid is roughly straight, and the posterior edge aligns with the posteriormost corner of the quadrate blade (Figure 17b).

The ectopterygoid overlaps the endopterygoid along its dorsomedial margin and is overlapped by the quadrate posterolaterally. It has a roughly oval shape (Figure 17).

The palatine is the anteriormost bone of the suspensorium. Its shaft is cylindrical, and its posterior end has a cartilaginous cap that articulates with the endopterygoid. A dorsolateral tubercle is positioned just anterior to the cartilaginous cap. The anterior end trifurcates into three processes directed dorsally, ventrally and laterally (Figures 1 and 19). The dorsal process is on the medial margin and narrows into a long, fine, dorsally directed tip. The ventral process is also medially positioned and is shorter and broader than the dorsal process, with a pointed shape. The dorsal and ventral processes form a deep groove over the posterolateral portion of the preethmoid (PE). The lateral process is laterally positioned and drawn into a broad, flat, wing that braces against the rostral process of the premaxilla (RP).

### 3.9 Oral jaws

**FIGURE 20:**
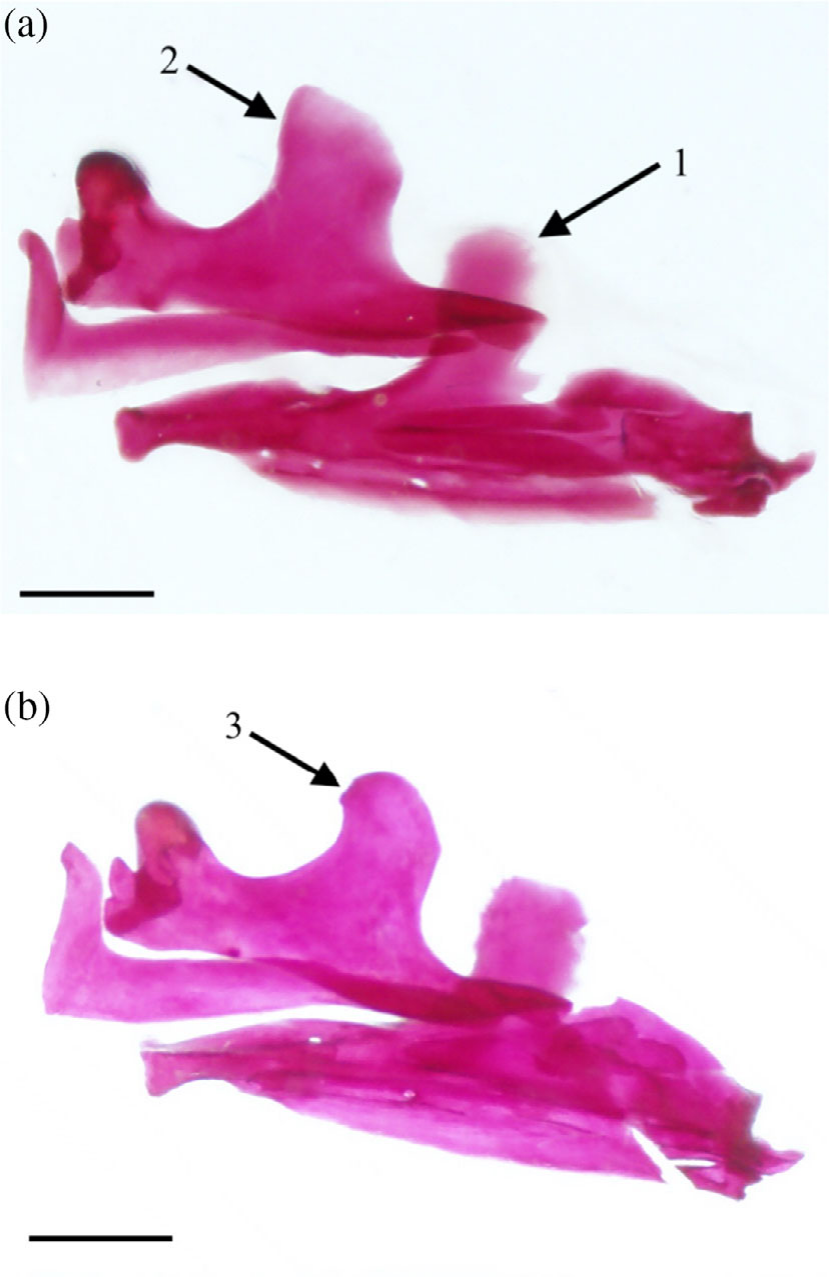
The left oral jaws of *Hudsonius hudsonius* UAMZ 213, specimen 5 (a) MZF 680, specimen 3 (b) in lateral view, anterior to left. Arrow 1 points to an elongate variant of the coronoid process of the dentary (compared with more robust coronoid process of the dentary in lower photograph). Arrow 2 points to the squared, pointed morphology of the ascending process of the maxilla found in other specimens. Arrow 3 points to the rounded ascending process of the maxilla found in some specimens. Specimens are in lateral view, anterior to left. Scale bars = 1 mm.

The dentary (DE) (Figure 17) is the largest bone of the lower jaw. Its anterior end curves medially to meet with its opposite. Its posterodorsal margin broadens into the flat, round, coronoid process. In nine sets of oral jaws examined, the coronoid process is narrower and more elongate than in the others (Figure 20a). The posterior margin of the dentary tapers to a point that terminates anterior to the retroarticular. The anteromedial margin is fused to the mentomeckelian (M), an ossification at the anterior end of the Meckel's cartilage. The entire anterior end of the anguloarticular (ART) slots into the posterior end of the dentary, with the Meckel's cartilage overlying the medial surfaces of both elements (Figure 17b).

The anguloarticular articulates with the retroarticular along its ventral margin (Figure 17). It has a deep socket at its posterior end, where the quadrate articulates. The posteroventral corner forms a blunt posterior process that extends under the anteroventral margin of the quadrate. The retroarticular is a small bone articulating with the anguloarticular along its dorsal margin. It has an irregular, elongate shape.

The coronomeckelian (CM) is found on the medial margin of the anguloarticular (Figure 17b). It is the extension of the ossification of the posterior end of the Meckel's cartilage. The coronomeckelian has a deep anterior notch and a central, medially directed flange.

The premaxilla (PMX) is slender, laterally curved and L‐shaped bone. It forms the entire gape of the upper jaw. A long, posteriorly concave ascending process contacts its opposite in the midline (Figure 1). The ascending process is broad at the base and tapers into a blunt point. The premaxilla tapers posteriorly into a laterally flattened shaft with a round end that curves ventrally.

The maxilla (MX) (Figures 1 and 17) is a laterally curved bone that overlaps and supports the premaxilla, acting as a lever to push the premaxilla as the mouth opens. The anterior end of the maxilla has two processes: the premaxillary process (PMP) and the rostral process posterior to it (Figure 1). The premaxillary process is short, flat and curves medially, overlapping the ascending process of the premaxilla when the mouth is closed (Figure 1). The rostral process is long and hook‐like and curves in medially where it is ligamentously joined to its opposite. Posterior to the rostral and premaxillary processes are two condyles, which serve as pivot points about which the maxilla rotates. When the mouth opens, the medial condyle braces against the preethmoid, whereas the lateral condyle braces against the lateral process of the palatine (LPP) (not illustrated in Figure 1). A small, pointed tubercle is observed along the anterolateral margin of the maxilla just posterior to the premaxillary process for the insertion of the adductor mandibulae. Posterior to the premaxillary process, the dorsal margin of the maxilla is deeply notched, creating a neck that leads to the ascending process. The ascending process of the maxilla is broad and flat with a round and anterodorsally oriented corner (Figure 20b). In 11 of the oral jaws examined, the ascending process is squared and drawn into a distinct, anterodorsal point (Figure 20a). In two sets of oral jaws examined, the ascending process is broadened and rectangular, whereas in one set, the ascending process is broad and round with a hooked anterodorsal corner. The posterior end of the maxilla tapers into a blunt end with a round posterior flange on the ventral margin. This posterior flange is overlapped by the posterior end of the premaxilla.

**FIGURE 21:**
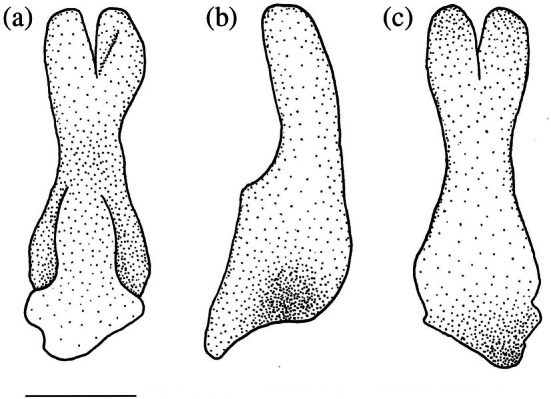
Illustration of the kinethmoid of *Hudsonius hudsonius* MZF 3215, specimen 1, in (a) anterior, (b) lateral and (c) posterior view when the mouth is closed, dorsal region to top of the page. Scale bar = 0.25 mm.

The kinethmoid (Figure 21) is a single, median endochondral element. When the mouth is closed, it lies between the maxillae, posterior to the premaxillae and anterior to the ethmoid block. The kinethmoid is roughly three times as tall as it is wide. The kinethmoid is constricted between dorsal and ventral portions. The dorsal portion of the kinethmoid is forked and curves anteriorly, whereas the ventral portion has a laterally positioned tubercle on either side and a posteriorly placed fossa that leads into a single, blunted, anteriorly positioned ventral process.

### 3.10 Circumorbital series 3.10

The circumorbital series surround the eye and include the supraorbital and the five infraorbitals (IO). The supraorbital is an ovular, dorsally curved element that lies along the dorsal margin of the eye (Figures 4 and 5). Its medial margin is convex, and its lateral margin is straight. In dorsal view, it is widest in the middle, narrowing posteriorly into a blunt end (Figure 5).

**FIGURE 22:**
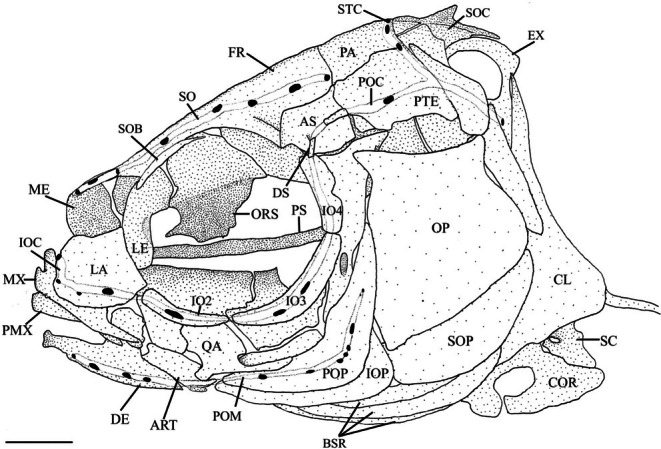
Illustration of the skull and sensory canal system of *Hudsonius hudsonius* UAMZ F1258, specimen 1, in left lateral view, anterior to left. ART, anguloarticular; AS, autosphenotic; BSR, branchiostegal rays; CL, cleithrum; COR, coracoid; DE, dentary; DS, dermosphenotic; EX, exoccipital; FR, frontal; IO, infraorbital; IOC, infraorbital canal; IOP; interopercle; LA, lacrimal; LE, lateral ethmoid; ME, mesethmoid portion of ethmoid block; MX, maxilla; OP, opercle; PA, parietal; PMX, premaxilla; POC, postocular commissure; POM, preopercular mandibular canal; POP, preopercle; PS, parasphenoid; PTE, pterotic; QA, quadrate; SC, scapula; SO, supraorbital canal; SOB, supraorbital; SOC, supraoccipital; SOP, subopercle; STC, supratemporal canal. Scale bar = 1 mm.

The infraorbitals are a series of five flat, plate‐like bones that surround the ventral and posterior margins of the orbit (Figure 22). The lacrimal (LA) is the first and largest element of the infraorbital series. It covers part of the nasal capsule and maxilla and is braced by the palatine. It is roughly as tall as it is wide, with five edges: dorsal, orbital, ventral, posterior and anterior. Overall, the lacrimal is roughly squared, but round anteriorly. In 14 of the specimens examined, the anterior edge is deepened. The ventral edge is convex and longer than the anterior edge. The posterior edge is short, abutting the second infraorbital.

The second infraorbital is elongate. The third infraorbital is longer and broader than the second, with a curved, crescentic shape. The fourth infraorbital lies along the posterior edge of the orbit and overlaps the anterior portion of the hyomandibula. The fifth infraorbital, or dermosphenotic (DS) (Coburn, 1982), overlies the autosphenotic spine and is reduced to a bony tube.

### 3.11 Sensory canal system

The sensory canals extend along and are present on some bones of the skull, oral jaws, opercular series and circumorbital series (Figure 22). The supraorbital canal (SO) extends through the nasal (N) and is enclosed in the frontal, extending along the dorsal margin of the orbit. The nasal portion usually has three pores, and the total number of pores is typically eight, but reaches nine in five specimens (Table 3). However, in three specimens, all from the same sample (MZF 2506), the nasal portion was absent, and the supraorbital portion had only six pores. The postocular commissure (POC), sometimes called the lateral temporal canal (Coburn, 1982), is enclosed in the pterotic and has a single pore. In one specimen, there are two pores instead of one (Table 3).

The supratemporal canal (STC) is present along the posteromedial margin of the parietal, extends to the pterotic and always has three pores. The preopercular mandibular canal (POM) is enclosed in and extends along the dentary, anguloarticular and preopercle. It typically has 10 pores, 4 from the dentary portion and 6 along the preopercular portion. The total number can range from 9 (four specimens) to 11 (five specimens) (Table 3). Pores along the dentary portion were observed to be as low as three (one specimen) and up to five (two specimens), and pores along the preopercular portion range from as low as five (four specimens) to as high as seven (four specimens) (Table 3).

The infraorbital canal (IOC) extends along the infraorbital series. There are typically seven pores: four on the lacrimal, one on the second infraorbital and two on the third infraorbital. The number of infraorbital pores can be as low as six (one specimen) and as high as eight (two specimens) (Table 3). One specimen has three pores on the lacrimal instead of four, whereas two specimens have three pores on the third infraorbital instead of the usual two.

### 3.12 Weberian apparatus

**FIGURE 23:**
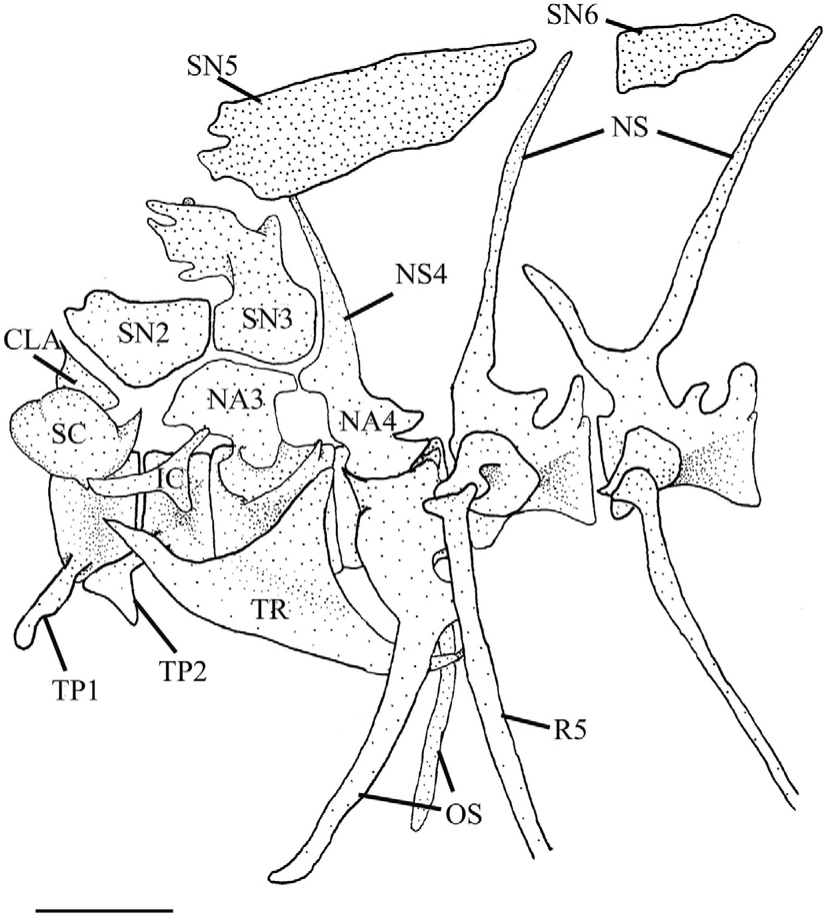
Illustration of the Weberian apparatus of *Hudsonius hudsonius* UAMZ 1258, specimen 1, in lateral view, anterior to left. CLA, claustrum; IC, intercalarium; TP, transverse process; NA, neural arch; NS, neural spine; OS; os suspensorium; R, rib; SC, scaphium; SN, supraneural; TR, tripus. Scale bar = 1 mm.

The Weberian apparatus (Figure 23) can be divided into two components: the pars sustentaculum and the pars auditum (Bird et al., 2020; Bird & Hernandez, 2007; Coburn, 1982). The pars sustentaculum is derived from the first four vertebrae, which have been modified to support the four Weberian ossicles, which form the pars auditum (Coburn, 1982).

#### 3.12.1 Pars sustentaculum

The first vertebra is a centrum with a transverse process (TP1) on either side projecting horizontally from the ventrolateral surface. The anterior face of the centrum articulates with the basioccipital.

The second vertebra is slightly smaller than the first. It has an amphicoelous centrum with large, blade‐like transverse processes (TP2) that project anterolaterally and extend under the anterior end of the transverse process of the first vertebra. The second supraneural is modified into a saddle‐shaped element that curves over the neural canal and has an anterior notch that the exoccipital fits into.

The third vertebra is the largest of the pars sustentaculum. It has an amphicoelous centrum with a small ventral lip that projects under the second vertebra. The posterolateral surface is grooved to support the foot of the tripus (TR). This groove is directed posterodorsally and develops into a ridge that extends slightly beyond the centrum. The third neural arch (NA3) is modified and can be broken down into three components. First is the base, which rests on the dorsal surface of the centrum. It is broader than the neck but narrower than the dorsal flange. The second is the neck, which is constricted and has its own expanded flange at its base. Third is the dorsal flange, which extends over the second vertebra, articulating with the second supraneural anteriorly, with the third supraneural (SN3) dorsally and with the fourth neural arch (NA4) posteriorly.

The third supraneural comprises a broad base, a constricted neck and a divided dorsal crest. The base of the third supraneural articulates with the second supraneural anteriorly, the third neural arch ventrally and the fourth neural spine (NS4) posteriorly. The crest of the third supraneural extends dorsally over the second vertebral centrum and the posterior portion of the second neural arch. In 20 of the Weberian apparatuses examined, the dorsal crest is elongate, projecting over more than half of the second neural arch. The double crest of the third supraneural is reduced and shortened in 10 of the Weberian apparatuses examined.

**FIGURE 24:**
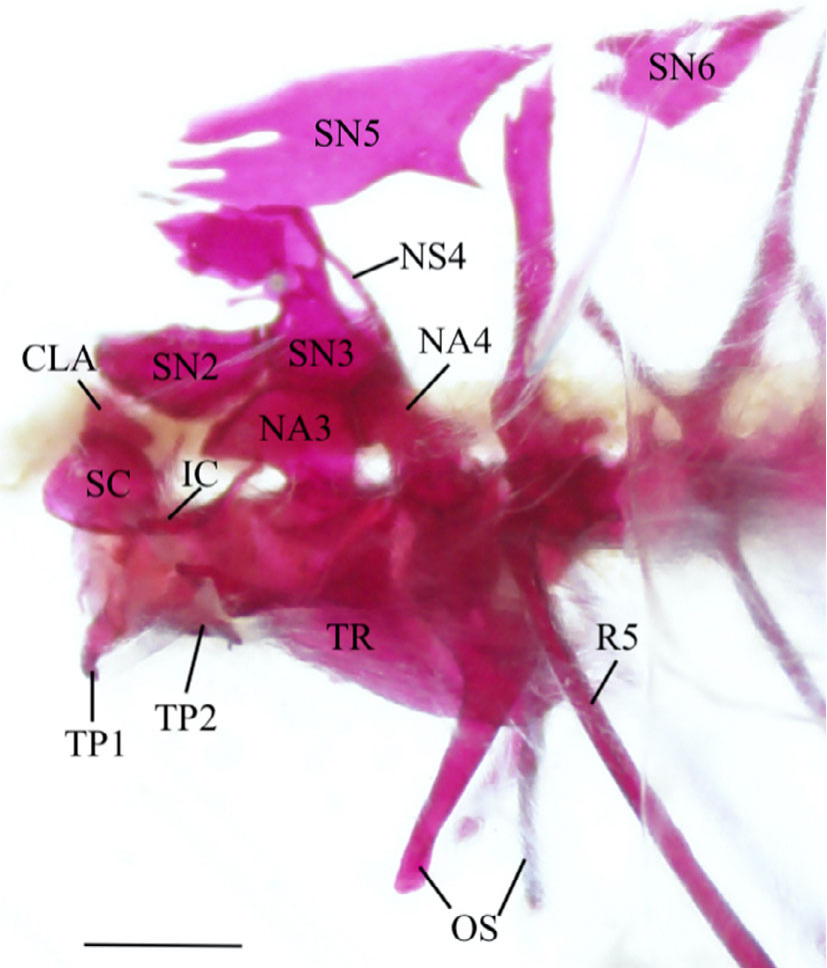
The Weberian apparatus of *Hudsonius hudsonius* UAMZ 4950, specimen 1, in lateral view, anterior to right. The fifth supraneural slots within the double crest of the third supraneural. CLA, claustrum; IC, intercalarium; TP, transverse process; NA, neural arch; NS, neural spine; OS, os suspensorium; R, rib; SC, scaphium; SN, supraneural; TR, tripus. Scale bar = 1 mm.

Five flat supraneurals lie dorsal to vertebrae 3–9. They are all irregularly shaped and become smaller posteriorly. The fifth supraneural (SN5) lies dorsal to the third, fourth and fifth vertebrae, above the dorsal crest of the third supraneural. The sixth supraneural (SN6) is found above the sixth vertebra. The seventh supraneural is found above the seventh vertebra; the eighth supraneural is found above the eighth vertebra; and the ninth supraneural is found above the ninth vertebra. In five of the Weberian apparatuses examined, the fifth supraneural fits between the left and right sides of the double crest of the third supraneural (Figure 24).

The fourth vertebra has an amphicoelous centrum with a modified neural arch and a neural spine (NS). The neural arch is autogenous with an expanded base and a deep, lateral groove that penetrates towards the base. It articulates with the third neural arch anteriorly and the third supraneural dorsally. The neural spine is shorter than those of the succeeding vertebrae and is directed anteriorly. The fourth vertebral centrum supports the os suspensorium, which comprises two arms. The outer arm is autogenous of the vertebral centrum and articulates with the fourth vertebral centrum via an expanded head and widens posterolaterally before tapering back anteriorly. The posteriormost corner of the outer arm lies under the fifth rib (R5). The inner arm of the os suspensorium is fused to the medial margin of the outer arm. It starts with a broad base that tapers and curves in medially, running in parallel to, but not articulating with, its opposite. The fifth vertebra is much like the other post‐Weberian vertebrae and differs only in its larger parapophysis and a thicker rib.

#### 3.12.2 Pars auditum

The claustrum (CLA) lies dorsal to the first vertebra and is overlapped by the scaphium (SC) laterally. The claustrum can be divided into a lower facet and an upper crest. The upper crest has a triangular shape, and the posterior edge is connected to the anteroventral edge of the second neural arch.

The scaphium rests along the posterolateral region of the first vertebra. It comprises a ‘cupped’ portion and separate, posteriorly placed dorsal and ventral processes. The cupped portion is the largest, with the opening on the medial side and a small tubercle on the middle of the lateral side. The dorsal process is dorsally directed with a thin, pointed end. The ventral process is anteriorly directed, is shorter than the ascending arm and attaches cartilaginously to the first vertebral centrum.

The intercalarium (IC) is a Y‐shaped ossicle with a single anterior arm and posterior articulating and ascending processes. The anterior arm is blunted, curving anterodorsally, and positioned lateral to the scaphium. The posterior end branches into the ascending and articulating processes. The ascending process is the longer of the two and is directed dorsally. The articulating arm is directed ventrally and articulates with the lateral surface of the second vertebral centrum.

The tripus is the largest Weberian ossicle. It has a dorsally concave shape. The tripus can be divided into a body with anterior and posterior arms. The body is triangular with a posterodorsally directed process. The tripus is positioned along the lateral side of the third vertebral centrum. The arms of the tripus are flattened. The anterior arm is shorter and broader, curving dorsomedially and terminating dorsal to the transverse process of the second vertebra and lateral to the anterior end of the intercalarium, to which it is ligamentously connected. A dorsal ridge originates from the medial edge of the anterior arm and runs dorsomedially to meet the dorsoposterior corner of the body. This ridge is absent in two of the Weberian apparatuses examined. The posterior arm runs posteroventrally, curling in medially and terminating between the inner and outer arms of the os suspensorium, connecting to the swim bladder.

### 3.13 Pectoral girdle

The posttemporal is a flat element that overlaps the supracleithrum ventrally. It is roughly tear‐dropped shaped, narrowing dorsally into a pointed end (Figures 29 and 30). Its anterodorsal margin contacts the pterotic, forming much of the roof and lateral margin of the posttemporal fossa.

The supracleithrum is a flat element overlapped by the posttemporal dorsally and overlapping the cleithrum (CL) ventrally (Figures 29 and 30). The dorsal margin has a blunt, hooked dorsal process. In five of the pectoral girdles examined, the dorsal process was straight, lacking the hook. The supracleithrum is widest at the base of the dorsal process and tapers into a blunt, round, ventral end. The posterodorsal corner of the opercle overlaps the anterodorsal margin of the supracleithrum.

**FIGURE 25:**
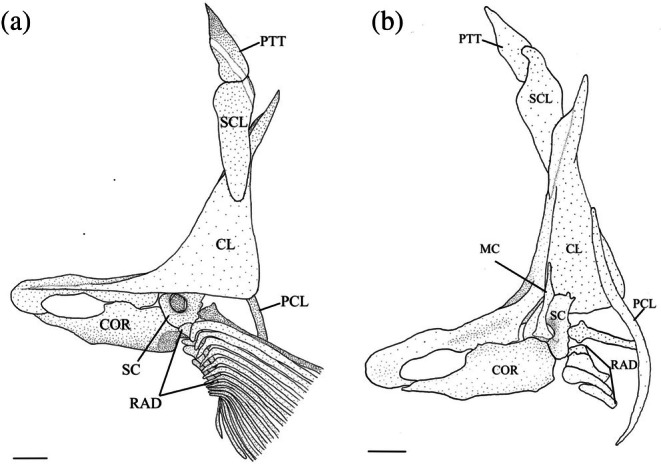
Illustration of the left pectoral girdle of *Hudsonius hudsonius* UAMZ 5644.1, specimen 1, in lateral (a) and medial (b) view, anterior to left (image b reversed). CL, cleithrum; COR, coracoid; RAD, radial; PCL, postcleithrum; PTT, posttemporal; SC, scapula; SCL, supracleithrum. Scale bars = 1 mm.

**FIGURE 26:**
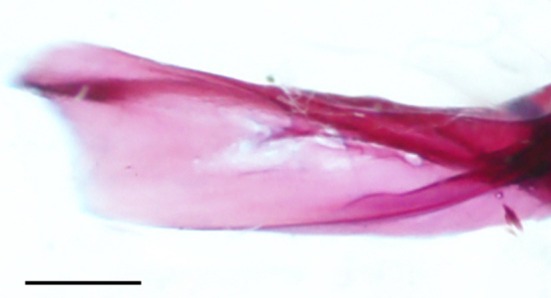
The horizontal arm of the cleithrum of *Hudsonius hudsonius* UAMZ 5644.1, specimen 1, in dorsal view, anterior to left. Scale bar = 1 mm.

The cleithrum is the largest bone of the pectoral girdle, with vertical and horizontal limbs of equal length that meet at an obtuse angle (Figure 25a). The anterior margin of the horizontal limb meets its opposite in the midline. The midpoint of the horizontal limb is narrowed, whereas the anterior and posterior margins broaden, and both articulate with the coracoid (COR) medially to create a large foramen. A distinct ridge runs along the medial surface of the horizontal limb, tapering posteriorly and terminating at the base of the vertical limb. When viewed dorsally, the anterolateral edge of the horizontal limb is shallowly concave (Figure 26). The vertical limb of the cleithrum consisted of a broad, triangular, lateral plate that starts along the anterior end of the horizontal limb and terminates at the end of the vertical limb in a blunt spine, and a medial ridge that widens dorsally and terminates into a moderately developed flange just before the dorsal spine of the lateral plate (Figure 25). The lateral plate of the vertical limb articulates with the lateral margin of the scapula (SC), whereas the mesocoracoid (MC) articulates with the anterior margin of the medial ridge of the horizontal limb of the cleithrum (Figure 25b).

**FIGURE 27:**
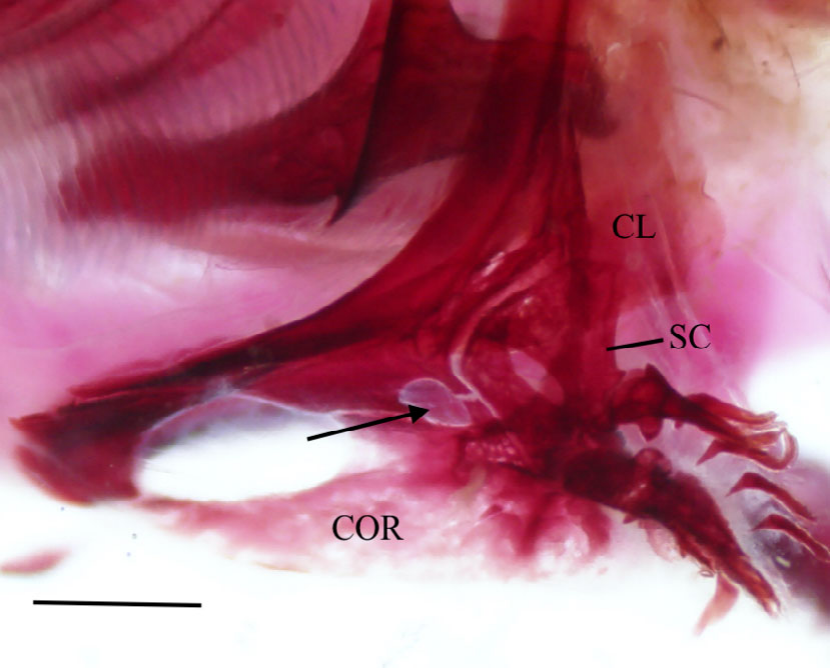
The left pectoral girdle of *Hudsonius hudsonius* UAMZ 221, specimen 4, in lateral view, anterior to left. Specimen shows secondary foramen formed by the coracoid and cleithrum (indicated by arrow). CL, cleithrum; COR, coracoid; SC, scapula. Scale bar = 1 mm.

The postcleithrum (PCL) is a thin, rod‐like, S‐shaped bone with pointed dorsal and ventral ends. Its dorsal end is attached to the posteromedial surface of the lateral plate of the cleithrum. The ventral end of the postcleithrum curves in medially (Figure 27).

The coracoid widens posteriorly, articulating with the cleithrum at its anterior and posterior ends, forming sutures. The posterior point of articulation is larger than the anterior. Where it articulates with the cleithrum, a large foramen is formed. The posterior end of the coracoid articulates with the scapula, whereas the posteromedial margin forms a bony shelf that articulates with the mesocoracoid. In one specimen, a unique pathology is observed in which the cleithrum and coracoid together form a second, anteriorly placed foramen (Figure 32).

The mesocoracoid (Figure 25b) is a triangular element that narrows dorsally. Its ventral margin articulates with the scapula posteriorly and the coracoid anteriorly. It has a round process along the anterodorsal margin that articulates with the median ridge of the ascending arm of the cleithrum.

**FIGURE 28:**
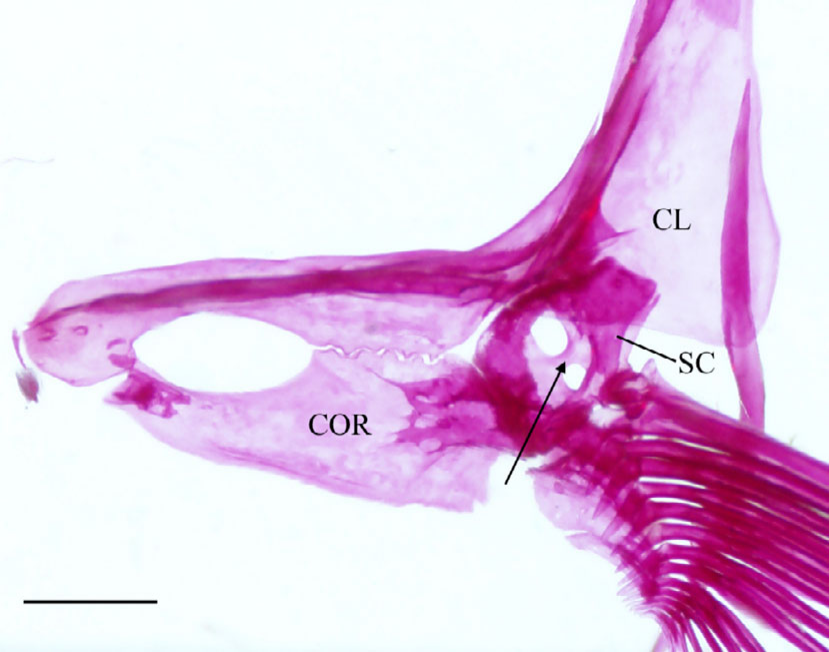
The left pectoral girdle of *Hudsonius hudsonius* UAMZ F1258, specimen 1, in lateral view, anterior to left. Specimen shows a scapular foramen divided by a bony strut (indicated by arrow). CL, cleithrum; COR, coracoid; SC, scapula. Scale bar = 1 mm.

The scapular foramen is large and positioned anterodorsally. In one of the pectoral girdles examined, the scapular foramen of the left scapula is divided into two by a bony strut (Figure 28). The dorsal margin of the scapula is round, articulating with the medial surface of the cleithrum. The posterior edge has a facet that articulates with the first pectoral‐fin ray. The posterodorsal corner of the scapula is developed into a weak, round flange that articulates with the medial surface of the cleithrum. In three of the pectoral girdles examined, the posterodorsal flange was strongly developed.

A total of four radials (RAD) support the rays of the pectoral fin. Counting dorsal to ventral, the first (dorsal most) radial is the shortest. It has a round shape and overlaps the posterolateral margin of the scapula. The second radial is broad and slightly shorter than the first and overlaps the medial margin of the scapula. The third radial is the longest, and the fourth is the thinnest.

**FIGURE 29:**
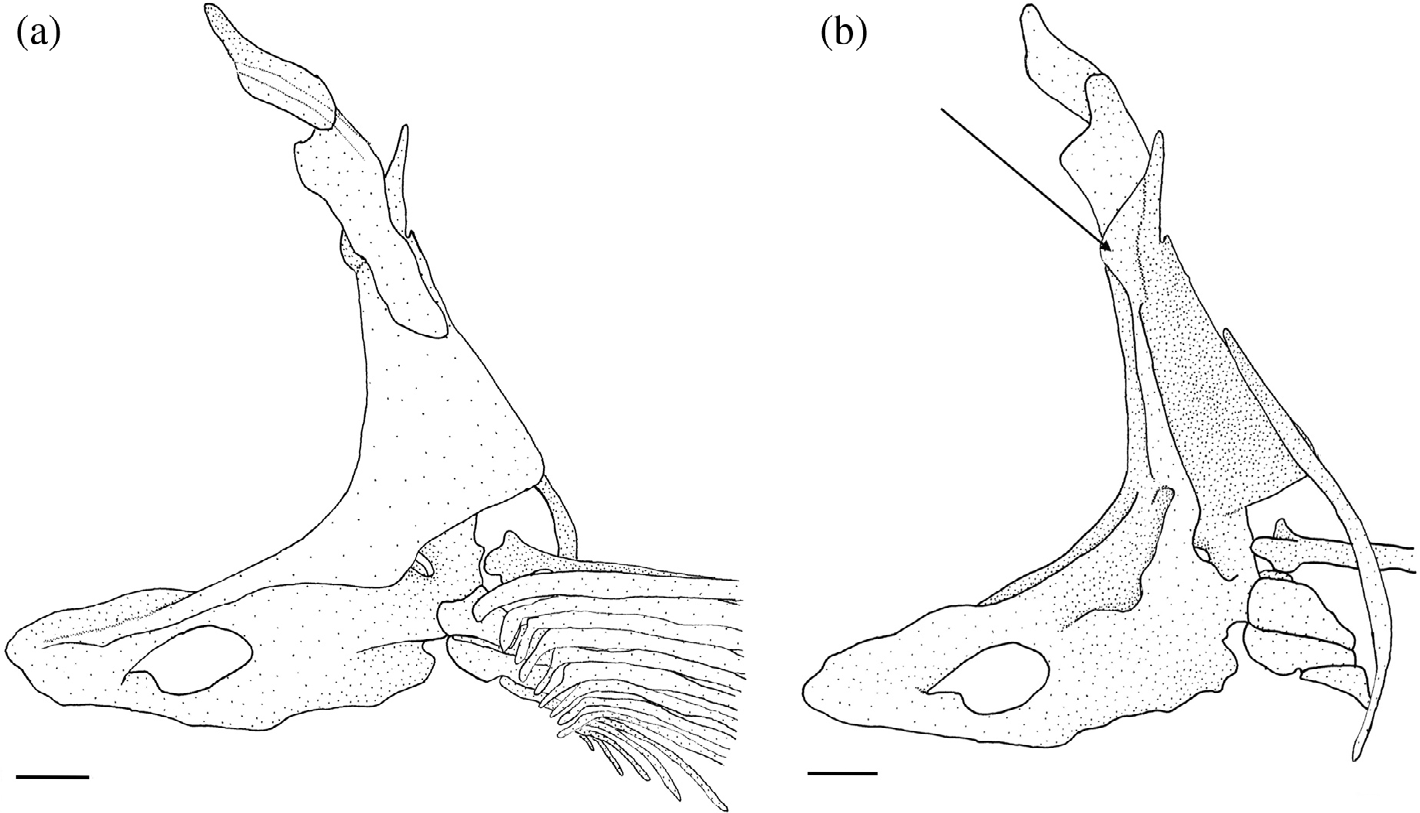
Illustration of the left pectoral girdle of *Hudsonius hudsonius* MZF 680, specimen 3, in lateral (a) and medial (b) view, anterior to left (image b reversed). Specimen shows fusion of the cleithrum, coracoid, mesocoracoid and scapula, and an enlarged dorsal flange on the medial ridge of the cleithrum (indicated by arrow). Scale bars = 1 mm.

In about 11 of the pectoral girdles I examined, there was complete fusion of the cleithrum, scapula, coracoid and mesocoracoid into a single element (Figure 29). These specimens, in addition to one that remained unfused, have an enlarged dorsal flange on the medial ridge of the cleithrum (Figure 29b). The dorsal spine of the lateral plate of the cleithrum was also noticeably shortened in these specimens (Figure 29b).

### 3.14 Pelvic girdle

**FIGURE 30:**
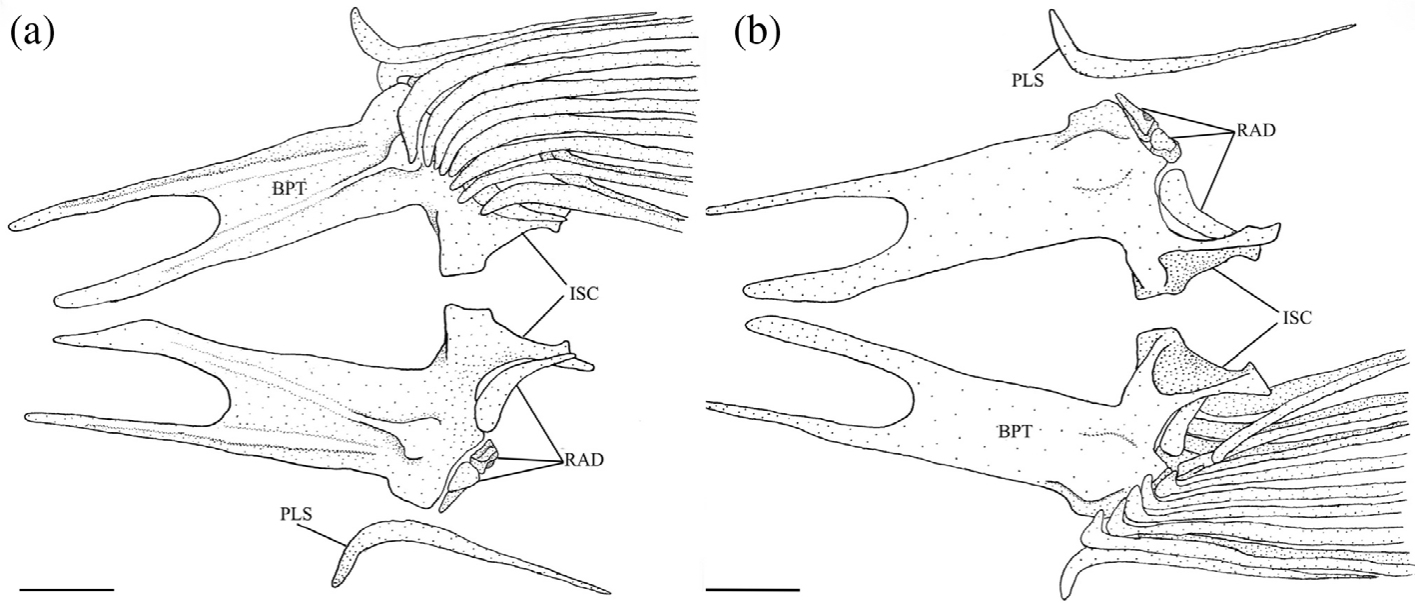
Illustration of the pelvic girdle of *Hudsonius hudsonius* UAMZ F9131, specimen 3, in dorsal (a) and ventral (b) view, anterior to left. The cartilage joining the basipterygia is not illustrated. BPT, basipterygium; ISC, ischiac process of basipterygium; PLS, pelvic splint; RAD, radial. Scale bars = 1 mm.

The pelvic girdle (Figure 30) consists of two basipterygia (BPT) that are joined by cartilage along their medial edges. Both basipterygia are dorsally concave and have forked anterior ends, producing two anteriorly projecting processes. The lateral process is longer and narrower than the medial process. In dorsal view, two shallow ridges run along the lateral and medial processes. The ridge of the lateral process extends farther up its length. A round tuberosity is observed at the base of the ridge of the medial process. When viewed ventrally, a weak tuberosity is observed at the posterior end of each basipterygium.

Each basipterygium has a broad, blunt, laterally curving ischiac process (ISC) found posterior to the point where the left and right sides are joined. Three radials support the rays of each pelvic fin. The medial‐most radial is a large, hooked element that curves medially and has an expanded base that is overlapped by the basipterygia ventrally. The middle and lateral radials are smaller, articulating along the posterolateral margin of the basipterygium. The lateral radial is larger than the middle radial. The pelvic splint (PLS) is an L‐shaped element that is tightly joined to the outer surface of the first pelvic ray. The anterior end of the pelvic splint has a short, anteroventrally directed ascending process.

### 3.15 Caudal skeleton

**FIGURE 31:**
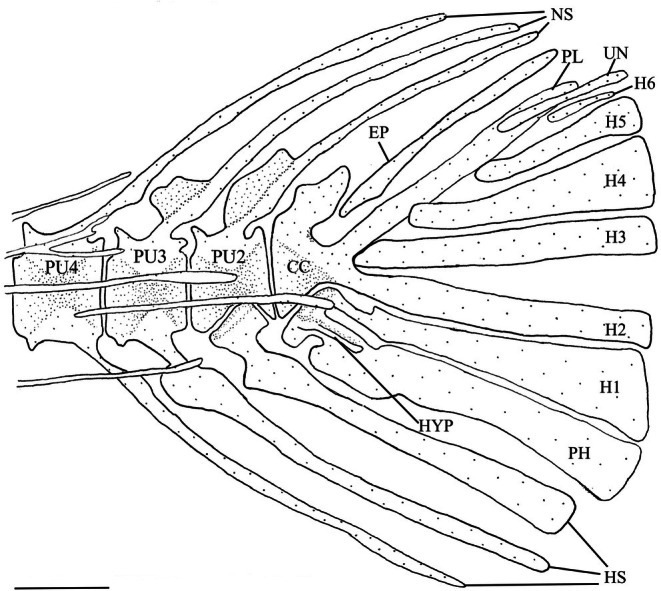
Illustration of the caudal skeleton of *Hudsonius hudsonius* UAMZ F9131, specimen 1, in left lateral view, anterior to left. CC, compound centrum; EP, epural; H, hypural; HS, hemal spine; HYP, hypurapophysis; NS, neural spine; PH, parhypural; PL, pleurostyle; PU, preural centrum; UN, uroneural. Scale bar = 1 mm.

**FIGURE 32:**
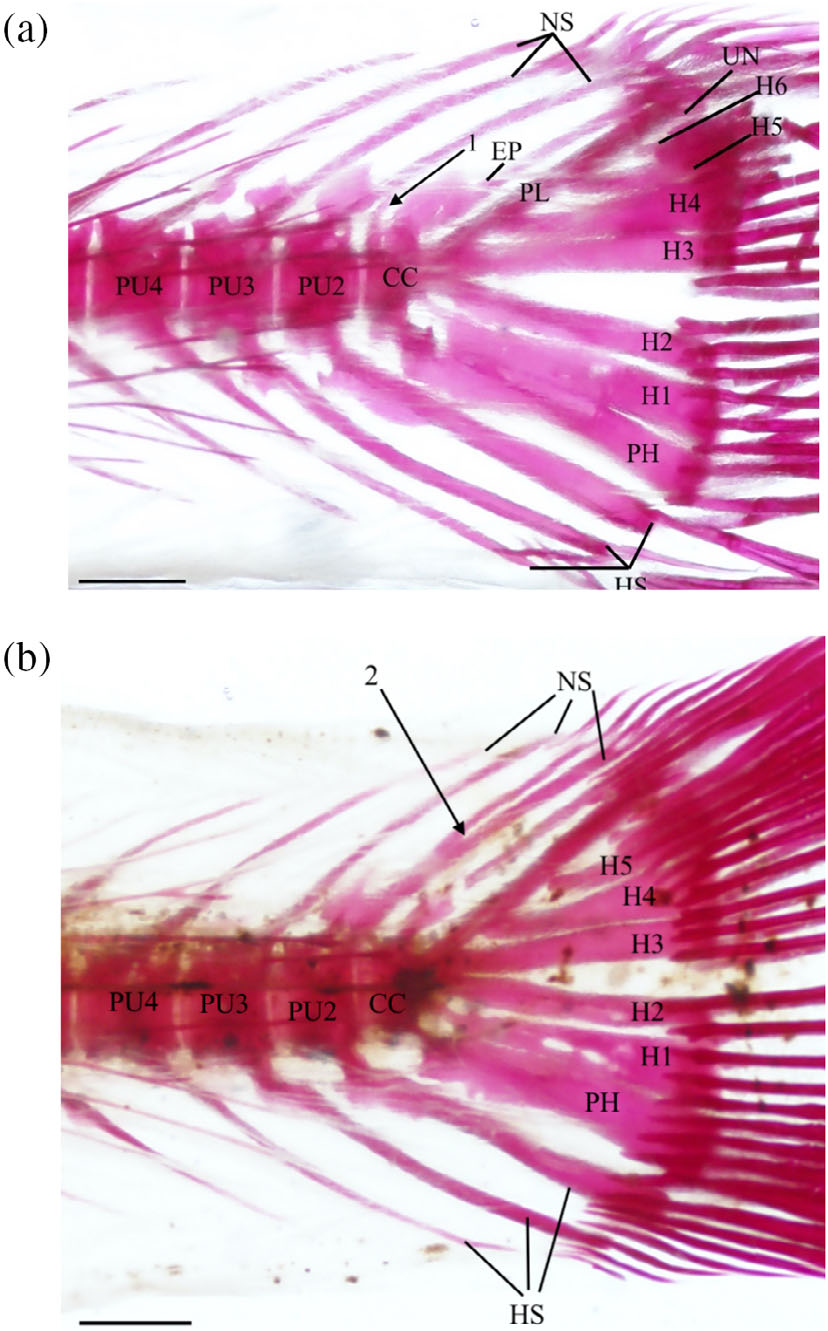
The caudal skeleton of *Hudsonius hudsonius* MZF 2506, specimen 1 (a), UAMZ F9126, specimen 2 (b) in left lateral view, anterior to left. Arrow 1 points to a secondary neural spine on the compound centrum. Arrow 2 points to a divided neural spine on the second preural centrum. CC, compound centrum; EP, epural; H, hypural; HS, hemal spine; HYP, hypurapophysis; NS, neural spine; PH, parhypural; PL, pleurostyle; PU, preural centrum; UN, uroneural. Scale bars = 1 mm.

**FIGURE 33:**
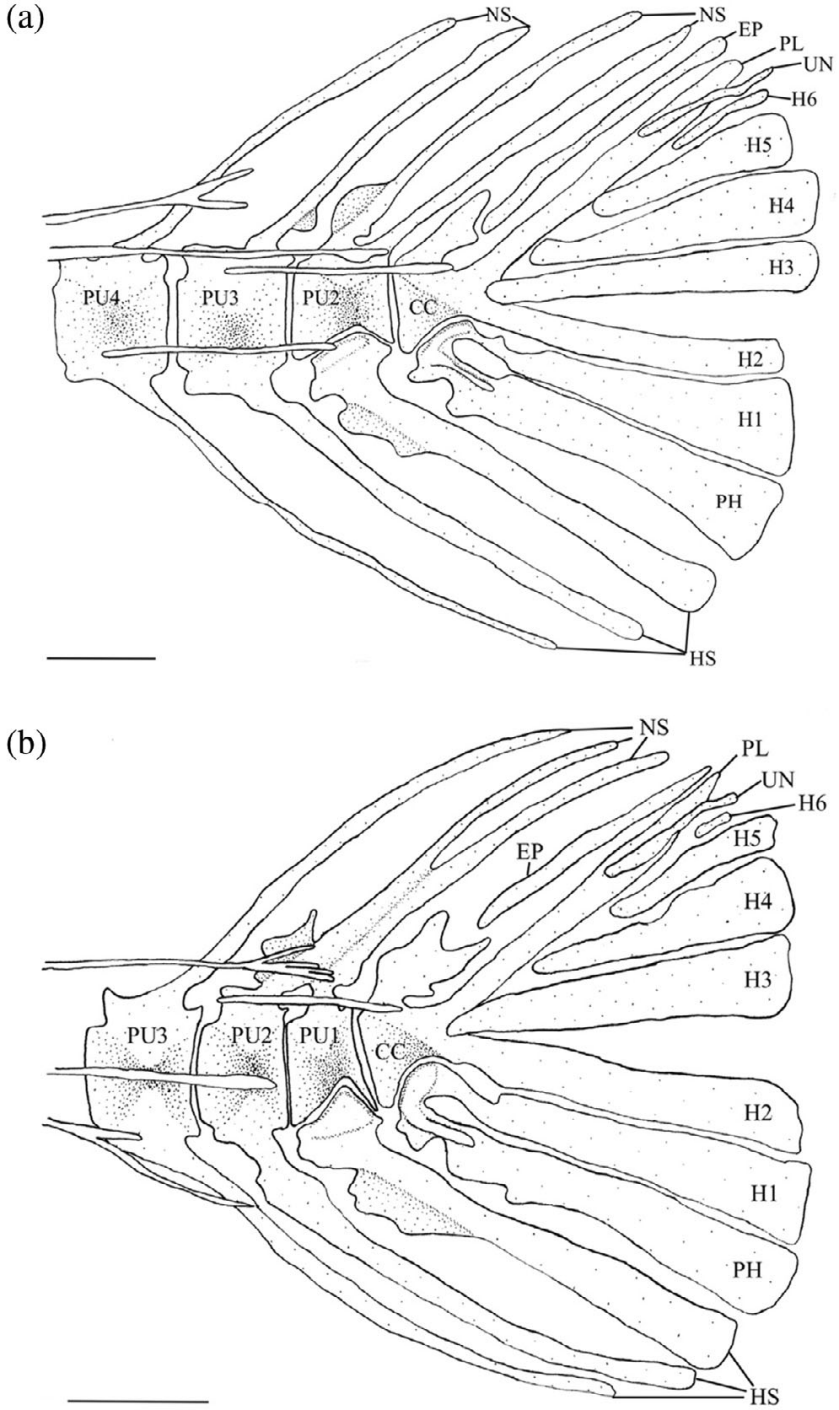
Illustration of the caudal skeleton of *Hudsonius hudsonius* UAMZ 3060, specimen 2 (a), UAMZ 209, specimen 1, (b) in left lateral view, anterior to left. Specimen (a) shows two neural spines on the preural centrum. Specimen (b) shows fusion of the neural spines of the second and third preural centra. CC, compound centrum; EP, epural; H, hypural; HS, hemal spine; HYP, hypurapophysis; NS, neural spine; PH, parhypural; PL, pleurostyle; PU, preural centrum; UN, uroneural. Scale bars = 1 mm.

**FIGURE 34:**
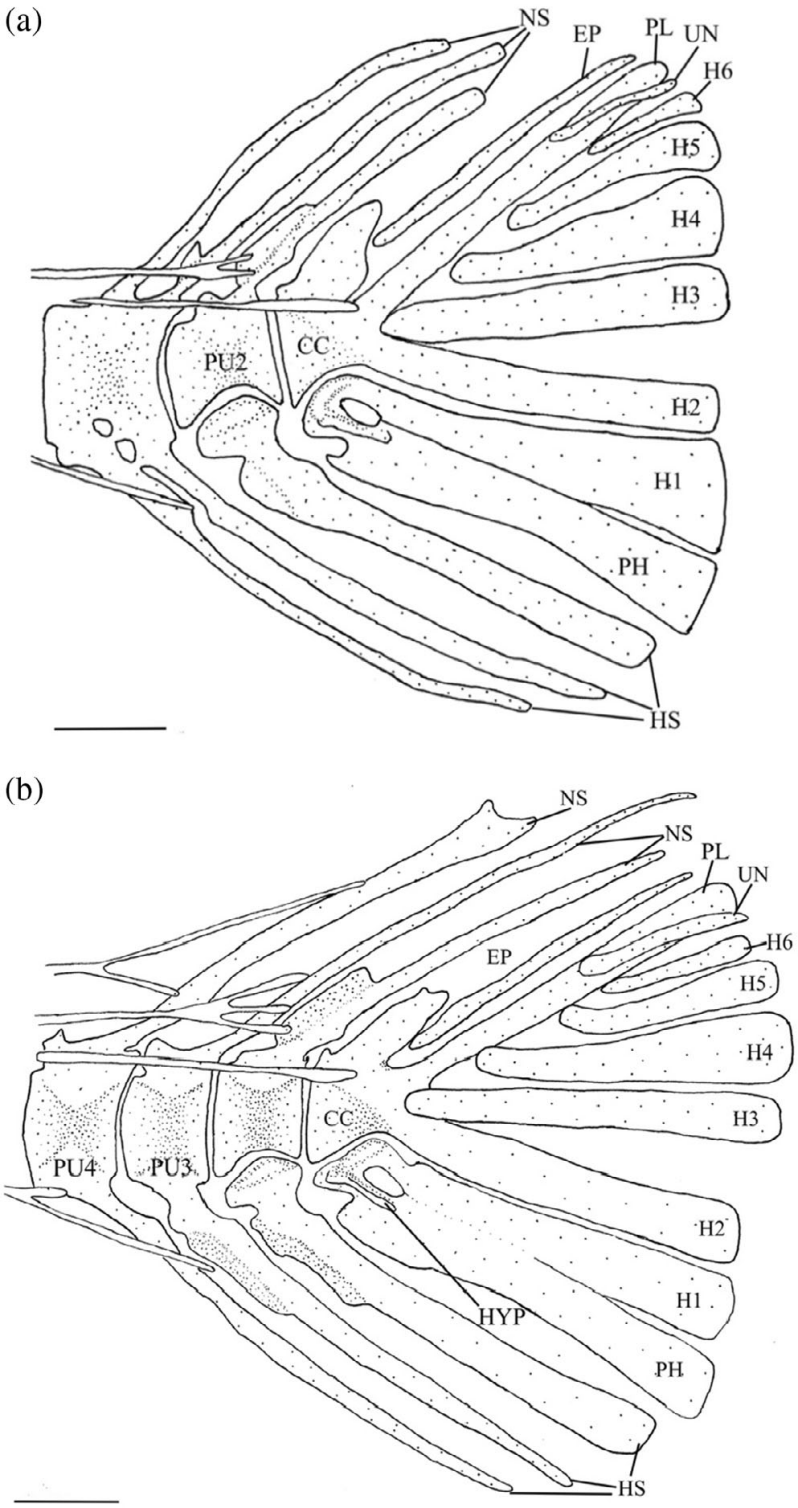
Illustration of the caudal skeleton of *Hudsonius hudsonius* UAMZ F9131, specimen 3 (a), UAMZ F9131, specimen 2 (b), in left lateral view, anterior to left. Specimen (a) shows abutting of the parhypural and first hypural and fusion of the third and fourth preural centra. Specimen (b) shows partial fusion of the parhypural and first hypural. CC, compound centrum; EP, epural; H, hypural; HS, hemal spine; HYP, hypurapophysis; NS, neural spine; PH, parhypural; PL, pleurostyle; PU, preural centrum; UN, uroneural. Scale bars = 1 mm.

As with other cyprinoids, the last four vertebrae of *H. hudsonius* (Figure 31) support the rays of the caudal fin (Buhan, 1972). The first preural vertebra is the compound centrum (CC), which supports an epural (EP), uroneural (UN), pleurostyle (PL), six hypurals (H1–H6) and a parhypural (PH). The compound centrum has a single, blunt, short neural spine. In one of the caudal skeletons examined, there are two neural spines on the compound centrum (Figure 32a). The following three vertebrae are preural centra, each with a single neural and hemal spine (HS). In seven of the caudal skeletons examined, there are two neural spines on the second preural centrum (PU2) (Figure 33a), whereas in three more caudal skeletons, there were two neural spines on the third preural centrum (PU3). In one caudal skeleton, there were two hemal spines on the third preural centrum, whereas in another, the neural spine of the second preural centrum was divided distally (Figure 32b). In another specimen, the neural spines of the second and third preural centra are fused together at the base (Figure 33b). In one specimen, the third and fourth preural centra (PU4) appear to be fused together, giving the appearance of an enlarged vertebra with two neural and hemal spines (Figure 34a).

The elements supporting the rays of the lower lobe of the caudal fin comprise the hemal spines of the second, third and fourth preural centra, the parhypural and the first and second hypurals. The hemal spine of the second preural centrum is autogenous. The parhypural and first hypural are fused together proximally and are together separate from the compound centrum. In 16 of the caudal skeletons examined, the parhypural and first hypural were partly fused together proximally (Figure 34b), whereas the parhypural and first hypural abutted but were not fused (Figure 34a) in eight. The first hypural has a thin, tapered base and widens distally. The parhypural bears a hypurapophysis (HYP). The second hypural is roughly rectangular and fused to the compound centrum. The hypurals and parhypural support the principal rays of the lower lobe of the caudal fin, whereas the ventral procurrent rays are supported by the hemal spines of the second, third and fourth preural vertebra.

**FIGURE 35:**
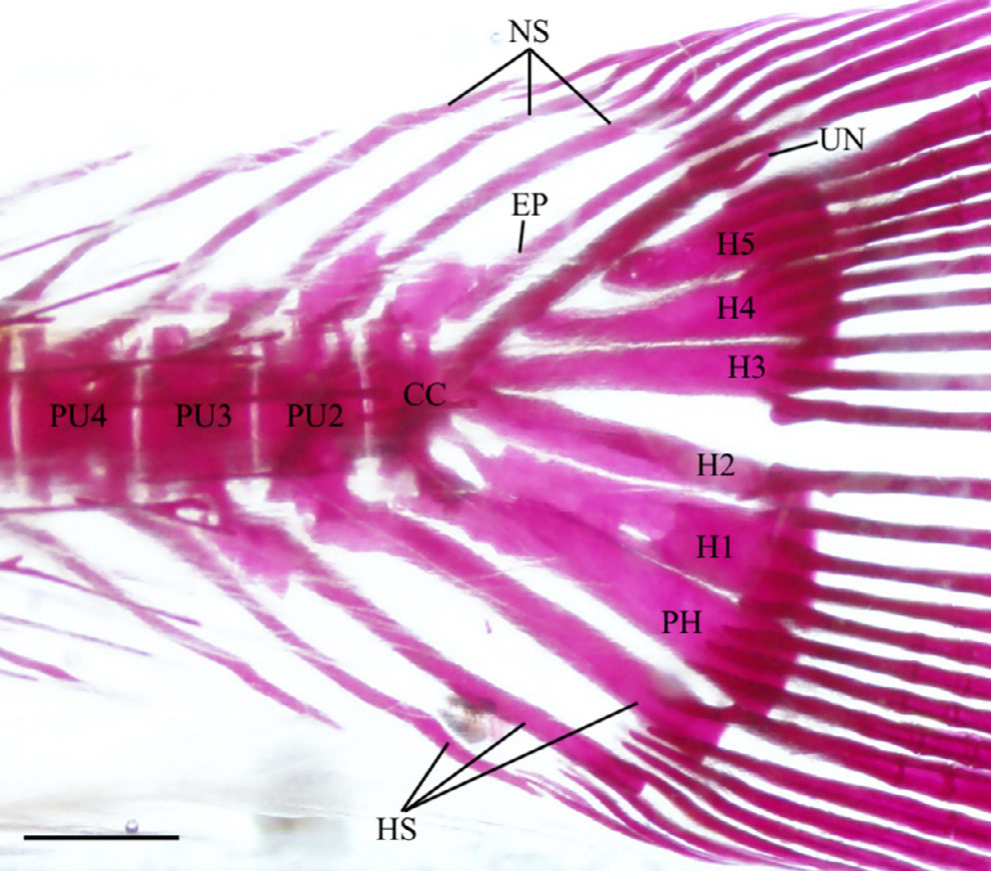
The caudal skeleton of *Hudsonius hudsonius* MZF 680, specimen 1, in left lateral view, anterior to left. Specimen shows fusion of the epural to the compound centrum, lack of the sixth hypural and an enlarged fifth hypural. CC, compound centrum; EP, epural; H, hypural; HS, hemal spine; HYP, hypurapophysis; NS, neural spine; PH, parhypural; PL, pleurostyle; PU, preural centrum; UN, uroneural. Scale bar = 1 mm.

The elements supporting the rays of the upper lobe of the caudal fin comprise the neural spines of the three preural centra plus the epural, pleurostyle, uroneurals and the third, fourth, fifth and sixth hypurals, with the sixth hypural always being the smallest. In one specimen, the sixth hypural is absent, and the fifth hypural is enlarged (Figure 35). The epural is a slender, elongate element that is almost always unfused to the compound centrum. In four of the caudal skeletons examined, the epural abutted but did not fuse with the compound centrum. In another two, the epural was fused to the compound centrum (Figure 35). The pleurostyle is broad and roughly squared and is firmly fused to the compound centrum. The uroneural is slender and is longer than the sixth hypural. The hypurals support the principal rays of the upper lobe, whereas the procurrent rays are supported by the uroneurals, epural and neural spines of the second, third and fourth preural vertebrae. There are consistently 19 caudal‐fin rays, 10 in the upper lobe and 9 in the lower lobe.

## 4 DISCUSSION

### 4.1 Measurements and meristic counts

There is a large degree of variation in the measurements and corresponding proportions of the *H. hudsonius* specimens used in this study. This variability can be observed both within and between *H. hudsonius* populations. In some samples (UAMZ 213, 214, 220 and 1525), there is a high degree of population‐level variability in measurements relating to peduncle length, predorsal length and body depth (Table 1).

**TABLE 1:** Measurements and ratios taken for *Hudsonius hudsonius* (*n* = 54).

Measurement	Minimum	Maximum	Mean	SD
Standard length (SL)	31.26	75.5	58.21	10.58
Pre‐anal length ${\mathrm{SL}}^{-1}$	0.63	0.77	0.7	0.03
Predorsal length ${\mathrm{SL}}^{-1}$	0.47	0.56	0.52	0.03
Pre‐pelvic length ${\mathrm{SL}}^{-1}$	0.46	0.56	0.52	0.03
Pre‐pectoral length ${\mathrm{SL}}^{-1}$	0.23	0.35	0.27	0.02
Head length (HL) ${\mathrm{SL}}^{-1}$	0.27	0.34	0.26	0.02
Snout length ${\mathrm{SL}}^{-1}$	0.03	0.15	0.07	0.02
Snout length ${\mathrm{HL}}^{-1}$	0.59	0.14	0.26	0.07
Postorbital length ${\mathrm{SL}}^{-1}$	0.08	0.14	0.11	0.01
Postorbital length ${\mathrm{HL}}^{-1}$	0.33	0.49	0.43	0.04
Dorsal‐fin base ${\mathrm{SL}}^{-1}$	0.10	0.18	0.14	0.02
Anal‐fin base ${\mathrm{SL}}^{-1}$	0.08	0.17	0.12	0.02
Peduncle length ${\mathrm{SL}}^{-1}$	0.17	0.30	0.23	0.03
Interorbital width ${\mathrm{SL}}^{-1}$	0.06	0.12	0.09	0.01
Interorbital width ${\mathrm{HL}}^{-1}$	0.23	0.43	0.35	0.05
Eye diameter ${\mathrm{SL}}^{-1}$	0.04	0.10	0.08	0.01
Eye diameter ${\mathrm{HL}}^{-1}$	0.18	0.39	0.30	0.05
Body depth ${\mathrm{SL}}^{-1}$	0.18	0.34	0.24	0.03
Caudal peduncle depth ${\mathrm{SL}}^{-1}$	0.06	0.13	0.10	0.01
Dorsal‐fin length ${\mathrm{SL}}^{-1}$	0.15	0.27	0.22	0.02
Anal‐fin length ${\mathrm{SL}}^{-1}$	0.11	0.21	0.18	0.02
Pectoral‐fin length ${\mathrm{SL}}^{-1}$	0.13	0.23	0.18	0.02
Pelvic‐fin length ${\mathrm{SL}}^{-1}$	0.11	0.19	0.16	0.02

*Note*: Measurements are presented in millimetres.

Of the meristic counts taken on the *H. hudsonius* specimens used in this study, the lateral line, predorsal scales and postdorsal scales had, by far, the largest ranges (Table 2). The circumferential scale count likewise has a larger range than most other meristic counts. The lateral‐line scale count of *H. hudsonius* has been reported to range from 38 to 42 (Scott & Crossman, 1973), 36 to 42 (Page & Burr, 2011) and 36 to 40 (Nelson & Paetz, 1992). However, the range of lateral‐line scales reported in this study (35–43) is still unprecedented. The lateral‐line scale count can vary as much within populations as it does between them. For example, in two samples, the lateral‐line scale count varied from 36 to 43 (UAMZ 214) and 37 to 42 (UMAZ 3215). Critically, there is no clear regional distinction between the eastern populations in Manitoba and Ontario and the western populations in Alberta and the Northwest Territories. A similar pattern is observed with respect to the circumferential, predorsal and postdorsal scale counts, reflecting a high degree of variability both between and within *H. hudsonius* populations. The range and frequency of the other meristic counts generally follow the observations of past researchers (Nelson & Paetz, 1992; Scott & Crossman, 1973). However, the gill raker counts that I took typically ranged from three to six, contrasting the range reported by Nelson and Paetz (1992) (four to nine). In one sample (UAMZ 1525), a single specimen had no gill rakers at all.

**TABLE 2:** Meristics for *Hudsonius hudsonius* (*n* = 54) unless otherwise stated).

Number									
Dorsal‐fin rays	7 (3)	8 (41)	9 (10)						
Anal‐fin rays	7 (3)	8 (45)	9 (6)						
Pelvic‐fin rays	7 (3)	8 (22)	9 (29)						
Pectoral‐fin rays (*n* = 53)	12 (2)	13 (6)	14 (28)	15 (12)	16 (5)				
Lateral‐line scales	35 (1)	36 (3)	37 (5)	38 (10)	39 (15)	40 (9)	41 (5)	42 (3)	43 (3)
Predorsal scales	12 (1)	13 (2)	14 (11)	15 (11)	16 (11)	17 (13)	18 (3)	19 (1)	20 (1)
Postdorsal scales	13 (1)	14 (3)	15 (4)	16 (8)	17 (13)	18 (12)	19 (11)	20 (1)	21 (1)
Scales above lateral line	4 (2)	5 (12)	6 (30)	7 (10)					
Scales below lateral line	4 (9)	5 (43)	6 (2)						
Circumferential scales	24 (2)	25 (9)	26 (18)	27 (11)	28 (8)	29 (6)			
Peduncle scales	10 (16)	11 (23)	12 (15)						
Gill rakers	0 (1)	3 (3)	4 (10)	5 (32)	6 (8)				
Predorsal vertebrae (*n* = 42)	10 (2)	11 (21)	12 (19)						
Total number of vertebrae (*n* = 42)	37 (5)	38 (22)	39 (10)	40 (4)	41 (1)				

*Note*: The number of specimens for each count is presented in brackets.

### 4.2 Intraspecific osteological variation in *H. hudsonius*

From this study, it is clear that *H. hudsonius* shows a high degree of osteological variability. Except for the pelvic girdle, I observed some form of intraspecific variation in almost all regions examined, though to varying degrees.

The sensory canal system of *H. hudsonius* mostly agrees with the descriptions of both Illick (1956) and Coburn (1982) regarding both structure and pore count (Table 3). However, the specimens from one sample (MZF 2506) showed a slight reduction in the supraorbital and infraorbital canals. In all three specimens examined, both the nasal portion of the infraorbital canal and the nasal bones themselves were absent. In one specimen, the infraorbital pore count was also reduced, with three lacrimal pores instead of the usual four. A reduction in the sensory canal system has been reported for other *Notropis* (s.l.) species as an adaptation to calm, low‐turbidity environments (Reno, 1966; Swift, 1970). This reduction is often observed in the supraorbital and infraorbital canals, as seen here. However, in these other species, when the supraorbital canal is reduced, the nasal portion is retained (Reno, 1966; Swift, 1970), contrary to what I observed in these *H. hudsonius* specimens, which were sampled from a lake environment. However, I did not see the same sensory reduction in specimens from other lakes. The loss of the nasal portion of the supraorbital canal is a phenomenon that will require further study.

**TABLE 3:** Sensory canal pore counts taken for *Hudsonius hudsonius* (*n* = 24).

Sensory canal				
Supraorbital	6 (3)	8 (16)		9 (5)
Supratemporal	3 (24)			
Postocular commissure	1 (23)	2 (1)		
Preopercular mandibular	9 (4)	10 (15)		11 (5)
Infraorbital	6 (1)	7 (21)		8 (2)

*Note*: The number of specimens that showed each count is presented in brackets.

Both the positioning of the hypohyal foramen and the shape of the urohyal are variable in *H. hudsonius*. Uyeno (1961) used the positioning of the hypohyal foramen, relative to the hypohyals and anterior ceratohyal, as a systematic character to distinguish different cyprinoid genera. The position and shape of the hypohyal foramen have also been featured in the descriptions of notropin species and genera (Coburn, 1982; Mayden, 1989) and in phylogenetic studies of North American cyprinoids (Coburn & Cavender, 1992). However, my analysis of *H. hudsonius* suggests that the exact position of the hypohyal foramen is variable. Although it is usually formed by the anterior ceratohyals and the dorsal and ventral hypohyals, it is restricted to the last two elements that represent a sizable minority, being observed in 18 of the 46 sets of hyoid bars examined. Rarely, the hypohyal foramen is formed only by the ventral hypohyal. Both character states are found in specimens from both the western and eastern populations. I frequently noted both character states in specimens from the same sample. The position of the hypohyal foramen is clearly variable within *H. hudsonius*. This variability should be considered when analysing other notropin species.

The urohyal shows two distinct variants regarding overall shape and wing proportions. One of these variants, in which the dorsal wing has a rounded, semicircular posterior edge that extends beyond the ventral wings, was observed only in specimens sampled from Alberta and the Northwest Territories. The other variant, where the dorsal wing is triangular and shorter than the ventral wings, is observed in specimens sampled from both Alberta, the Northwest Territories, Manitoba and Ontario. Although the second variant was by far the most common in the western populations, a mixture of both variants is often observed in specimens from a single sample. The second variant was prevalent in the three samples collected from Manitoba and western Ontario. However, one urohyal from a specimen sampled from Ontario had a distinct shape: the ventral wings were much longer than the dorsal wing, and the posterior margin of the dorsal wing was almost straight. In his study on the evolutionary relationships of North American cyprinoids, Mayden (1989) noted that roughly 40% of the *H. hudsonius* specimens examined had a pair of lateral processes at the base of the neck of the urohyal. However, I did not observe this specific variant in the *H. hudsonius* specimens that I examined.

The branchial apparatus of *H. hudsonius* is unique in that the third epibranchial has a dorsal process, a trait that is not described in any other notropin species (Coburn, 1982; Mayden, 1989). Similarly, the variable presence and absence of the dorsal process on the fourth epibranchial contradict the descriptions of other notropin species, with the dorsal process always present (Coburn, 1982; Mayden, 1989). Although documented in only one specimen, the presence of four basibranchials instead of three is a unique variant not previously reported in the literature. Given the rarity of this variant, it is likely either a pathological condition or morphological variation that evolved only in the population from which the specimen was sampled (MZF 680). The usual dental formula (1,4–4,1) that I observed in *H. hudsonius* contrasts with Eastman and Underhill (1973) and Hubbs and Lagler (2004), who found the usual formula of 2.4–4.2. In contrast, Peer (1961) and Nelson and Paetz (1992) reported the dental formula of *H. hudsonius* to be variable, between 1.4–4.1 and 2.4–4.2. However, Eastman and Underhill (1973) observed the same variety of dental formulas in *H. hudsonius* that I observed myself (Table 3), with the exception of 0.3–4.0.

In their analysis of 42 notropin species native to Minnesota, Eastman and Underhill (1973) noted that the dental formula of *H. hudsonius* was by far the most variable among all the species studied. Most of this variation was because of a missing tooth on the minor row of one arch. The authors concluded that *H. hudsonius* was evolving towards a 1.4–4.1 condition. The results of my own analysis may reflect this trend. However, it should be noted that the *H. hudsonius* specimens used by Eastman and Underhill (1973) were sampled in Minnesota and South Dakota. By comparison, my specimens were sampled in Alberta, Manitoba, Ontario and the Northwest Territories. Thus, this difference in dental formula could also reflect regional differences among *H. hudsonius* populations.

The pelvic girdle shows no notable variants beyond pelvic‐fin ray count. In contrast, the pectoral girdle shows a few notable variations. The fusion of the coracoid, mesocoracoid, cleithrum and scapula into a single element is a novel variant not previously reported by other researchers. This fused variant was predominant in specimens sampled from Manitoba and western Ontario, with eight out of nine specimens showing this variant. This fusion is almost always accompanied by an enlarged dorsal flange on the medial ridge of the cleithrum and a shortened dorsal spine of the lateral plate of the cleithrum. Excluding specimens sampled from Manitoba and Ontario, I observed the fused variant only three times in specimens from Alberta. Two of these specimens also exhibited an enlarged dorsal flange on the medial ridge of the cleithrum and a shortened dorsal spine. Three other specimens showed partial fusion of the pectoral girdle, one from Alberta and the other two from the Northwest Territories, and also had an enlarged dorsal flange and shortened dorsal spine. In another specimen, also from Alberta, the pectoral girdle was unfused, but the cleithrum had the same enlarged flange and dorsal spine typically observed in fused pectoral girdles. In yet another specimen, the pectoral girdle was fused, but the dorsal flange was reduced, and the dorsal spine elongate, as typically seen in unfused pectoral girdles. My results suggest that this fused variant is much more prevalent in eastern *H. hudsonius* populations, but not isolated to them. However, the frequency of this fused variant clearly differs between the eastern and western regions.

*H. hudsonius* consistently has 19 caudal‐fin rays, 10 in the upper lobe and 9 in the lower, a plesiomorphic condition in ostariophysians and common among Cypriniformes (Coburn, 1982; Lundberg & Baskin, 1969). However, the caudal skeleton itself shows variability. The most notable variant is the fusion of the parhypural with the first hypural distally. In one specimen, there is a loss of the sixth hypural and an enlarged fifth hypural. Fusion and loss of the hypurals have been noted in North American cyprinoids, usually as a species‐specific trait (Buhan, 1972; Mayden, 1989). However, the fusion of the first hypural and parhypural seems to be unique to *H. hudsonius* (Buhan, 1972). Of the caudal skeletons I examined, fusion of the parhypural and first hypural was the most common variation compared to most specimens in which these two bones are only fused proximally at the base. However, it is not uncommon to have the parhypural and first hypural abut distally, but not fuse. All specimens sampled from Manitoba and Ontario show either fusion or abutting of the parhypural and first hypural. These specimens were also on the larger side, ranging between 63.2 and 75.5 mm in standard length. However, I observed the unfused state in specimens of similar size sampled from western populations. I also noted fusion of the parhypural and first hypural in smaller specimens that were under 60 mm in standard length. Thus, I suspect the fusion of the parhypural and first hypural to be linked to population and region rather than body size. Other variations in the caudal skeleton that I observed, including two neural or hemal spines on a preural centrum, division of a neural spine and fusion of two preural centra are, based on their rarity, most likely individual variants that are of no taxonomic significance.

Variations observed in the suspensorium, oral jaws and infraorbital series relate to the shape of a few specific elements. For the suspensorium, it is the shape of the anterodorsal process of the metapterygoid and the anterior wing of the hyomandibula. Past descriptions of other notropin, and indeed cyprinoid, species always mention that a distinct, well‐developed adductor ride is present on the lateral side of the hyomandibula at the level of the opercular condyle (Buhan, 1970; Coburn, 1982; Mayden, 1989; Uyeno, 1961). However, I observed a weakly developed adductor ridge in only four of the suspensoria examined and a well‐developed adductor ridge in only one specimen. For the oral jaws, variation is observed in the shape of the ascending process of the maxilla and coronoid process of the dentary. For the infraorbital series, it is the anterior edge of the lacrimal. These variants are not isolated to any one population or region. However, the fact that these kinds of variations can exist should be acknowledged when examining and comparing other notropin species.

For the Weberian apparatus, specimens sampled from the Northwest Territories consistently have an elongate dorsal crest that projects over more than half of the second neural arch. However, this variant is observed in other populations, though less frequently. In samples taken from two populations in east central Alberta (UAMZ 213, 4950), the first predorsal bone fits between the left and right sides of the double crest of the third supraneural in five specimens. Otherwise, variations in the Weberian apparatus are not generally associated with a specific population or region.

Coburn (1982) noted four foramina in the pterosphenoid, in contrast with Harrington (1955), who reported only two. My own observations align with Coburn (1982). Coburn (1982) noted that the foramen for the ophthalmic branch of the trigeminal nerve was often continuous with the trigeminal foramen instead of separated. However, I did not observe this variant in *H. hudsonius*. However, I did observe that the position of the olfactory, trigeminal and oculomotor foramina appears to be variable. Though rare, the olfactory foramen was sometimes formed exclusively within the lateral ethmoid instead of being formed by the mesethmoid anteriorly and the lateral ethmoids posteriorly as is common in the specimens that I analysed. Similarly, the trigeminal foramen, while usually formed by both the pterosphenoid and prootic, is in one instance formed only by the prootic. In three specimens, it is formed by the prootic on only one side and the pterosphenoid and prootic on the other. The oculomotor foramen is more variable, being formed between the prootics and pterosphenoids or only the prootics. These variants are also not restricted to any single population or region. However, these types of variants should be considered when examining and comparing other notropin species.

Coburn (1982) and Mayden (1989) described what has since been termed the open posterior myodome (OPM). The OPM is an opening bounded by the parasphenoid anteriorly and the basioccipital posteriorly. Coburn (1982) was the first to describe the OPM. Mayden (1989) went further by suggesting that the OPM was a synapomorphy shared by all North American cyprinoids. He also noted a secondary closure in the genera *Nocomis* Girard, 1856, *Campostoma* Agassiz, 1855 and *Dionda* Girard, 1856 and the species *Playygobio gracilis* Richardson, 1836, *Notropis boucardi* Günther, 1868 and *Notropis bifrenatus* Cope, (1867). In these taxa, Mayden (1989) observed that the OPM was present in juveniles but fused shut in adults. He considered this secondary closure to be a derived trait. All other North American cyprinoids examined by Mayden (1989) lacked this secondary closure, including *H. hudsonius*. Contrary to Mayden (1989), I did not observe an OPM in any of the *H. hudsonius* specimens examined. Instead, I observed a secondary closure (Figure 4), the CPM.

### 4.3 Conclusions

From this analysis, *H. hudsonius* is clearly a species with a high degree of intraspecific variation. Some osteological variants are observed in only a few specimens, whereas others are observed more frequently in individuals from the 15 populations I examined. A few variants seem to reflect regional differentiation based on what variants are most common in specimens from different regions. Overall, three osteological characteristics appear to vary in frequency between western populations in Alberta and the Northwest Territories and eastern populations in Manitoba and Ontario. The first is the fusion of the cleithrum, coracoid, mesocoracoid and scapula into a single element, accompanied by the presence of an enlarged dorsal flange on the medial ridge of the cleithrum and a shortened dorsal spine, which is found predominantly in eastern populations and rarely observed in western populations. The second is the dorsal wing of the urohyal, which, in lateral view, is usually triangular and shorter than the ventral wings. However, it can also be rounded and slightly longer than the ventral wings in western populations. The third is the caudal skeleton. In western populations, the parhypural and first hypural are usually fused proximally at the base. Rarely, there is fusion or abutting of the parhypural and first hypural distally. In contrast, the fusion or abutting of the parhypural and first hypural is consistently observed in eastern populations.

These variations in the osteological characters examined raise further questions about the regional differentiation within *H. hudsonius* as a species. Furthermore, I would suggest that these findings indicate that there may be specific or subspecific differences among these populations and regions. However, the specimens examined in this study are from the western portion of the range traditionally attributed to *H. hudsonius* (Page & Burr, 2011). To determine if these populations should be recognized as distinct species or subspecies, more research will need to be done. This might include a study that focuses on the osteology of *H. hudsonius* populations in eastern Canada and the US. Further research will also need to focus on the genetic diversity of *H. hudsonius* to see if genetic differences between eastern and western populations correlate with the osteological differences I have observed. Genetic‐based research should more broadly focus on potential genetic differences of *H. hudsonius* throughout its native range. Both osteological and genetic analyses will be necessary to understand the evolution and regional diversity of *H. hudsonius*, and whether this species, as it is currently classified, can be further divided into subspecies or even species.

## AUTHOR CONTRIBUTIONS

Erika K. Jessen cleared and stained all specimens, dissected specimens, took photographs, did specimen drawings and wrote the whole manuscript.

## FUNDING INFORMATION

Funding for this work was provided by the University of Alberta.
